# Recommendations for the Design and Delivery of Transitions-Focused Digital Health Interventions: Rapid Review

**DOI:** 10.2196/35929

**Published:** 2022-05-19

**Authors:** Hardeep Singh, Terence Tang, Carolyn Steele Gray, Kristina Kokorelias, Rachel Thombs, Donna Plett, Matthew Heffernan, Carlotta M Jarach, Alana Armas, Susan Law, Heather V Cunningham, Jason Xin Nie, Moriah E Ellen, Kednapa Thavorn, Michelle LA Nelson

**Affiliations:** 1 Department of Occupational Science & Occupational Therapy Temerty Faculty of Medicine University of Toronto Toronto, ON Canada; 2 March of Dimes Canada Toronto, ON Canada; 3 Rehabilitation Sciences Institute Temerty Faculty of Medicine University of Toronto Toronto, ON Canada; 4 Toronto Rehabilitation Institute University Health Network Toronto, ON Canada; 5 Institute for Better Health Trillium Health Partners Mississauga, ON Canada; 6 Department of Medicine University of Toronto Toronto, ON Canada; 7 Collaboratory for Research and Innovation Lunenfeld-Tanenbaum Research Institute Sinai Health System Toronto, ON Canada; 8 Institute of Health Policy, Management and Evaluation Dalla Lana School of Public Health University of Toronto Toronto, ON Canada; 9 St. John’s Rehab Research Program Sunnybrook Research Institute Sunnybrook Health Sciences Centre Toronto, ON Canada; 10 Department of Environmental Health Sciences Istituto di Ricerche Farmacologiche Mario Negri IRCCS Milan Italy; 11 Gerstein Science Information Centre University of Toronto Toronto, ON Canada; 12 Department of Health Policy and Management Guilford Glazer Faculty of Business and Management and Faculty of Health Sciences Ben-Gurion University of the Negev Beer-Sheva Israel; 13 Clinical Epidemiology Program Ottawa Hospital Research Institute Ottawa, ON Canada; 14 School of Epidemiology and Public Health University of Ottawa Ottawa, ON Canada

**Keywords:** transitions, health, medical informatics, aged, mobile phone

## Abstract

**Background:**

Older adults experience a high risk of adverse events during hospital-to-home transitions. Implementation barriers have prevented widespread clinical uptake of the various digital health technologies that aim to support hospital-to-home transitions.

**Objective:**

To guide the development of a digital health intervention to support transitions from hospital to home (the Digital Bridge intervention), the specific objectives of this review were to describe the various roles and functions of health care providers supporting hospital-to-home transitions for older adults, allowing future technologies to be more targeted to support their work; describe the types of digital health interventions used to facilitate the transition from hospital to home for older adults and elucidate how these interventions support the roles and functions of providers; describe the lessons learned from the design and implementation of these interventions; and identify opportunities to improve the fit between technology and provider functions within the Digital Bridge intervention and other transition-focused digital health interventions.

**Methods:**

This 2-phase rapid review involved a selective review of providers’ roles and their functions during hospital-to-home transitions (phase 1) and a structured literature review on digital health interventions used to support older adults’ hospital-to-home transitions (phase 2). During the analysis, the technology functions identified in phase 2 were linked to the provider roles and functions identified in phase 1.

**Results:**

In phase 1, various provider roles were identified that facilitated hospital-to-home transitions, including navigation-specific roles and the roles of nurses and physicians. The key transition functions performed by providers were related to the 3 categories of continuity of care (ie, informational, management, and relational continuity). Phase 2, included articles (n=142) that reported digital health interventions targeting various medical conditions or groups. Most digital health interventions supported management continuity (eg, follow-up, assessment, and monitoring of patients’ status after hospital discharge), whereas informational and relational continuity were the least supported. The lessons learned from the interventions were categorized into technology- and research-related challenges and opportunities and informed several recommendations to guide the design of transition-focused digital health interventions.

**Conclusions:**

This review highlights the need for Digital Bridge and other digital health interventions to align the design and delivery of digital health interventions with provider functions, design and test interventions with older adults, and examine multilevel outcomes.

**International Registered Report Identifier (IRRID):**

RR2-10.1136/bmjopen-2020-045596

## Introduction

### Background

Hospital-to-home transitions can be a challenging time for older adults [1-10] owing to the high risk of adverse events, including medical errors, hospital readmission, and death [4,7,11,12]. It has been noted that almost half of the adverse events experienced during these transitions could be prevented or minimized [4,7,11,12]. Furthermore, pressures facing health care systems have resulted in decreased lengths of hospital stay, leading to patients being discharged *quicker and sicker* and an increased risk of hospital readmissions and poor health outcomes [13-16]. The costly and negative impacts of poor transitions have made transitions a high priority for the health care system and prompted significant efforts to improve hospital-to-home transitions [17].

Multidisciplinary teamwork is one of the critical aspects of high-quality continuity of care [18]. Facilitating successful hospital-to-home transitions involves team effort because multiple tasks must be completed by various health care providers across inpatient and community settings [16]. Information-sharing and communication issues combined with a lack of role clarity can cause poor continuity of care and service fragmentation during transitions [16,19-22].

Improving hospital-to-home transitions entails improving communication and coordination among multiple providers and across multiple health care settings [23,24]. Rennke and Ranji [17] have suggested that successful hospital-initiated transitional care programs include a “bridging” strategy with pre- and postdischarge interventions. Although numerous transitional care models and strategies have been proposed [17,25-31], they require considerable resources, such as a dedicated transition provider, because of the additional work required [16,17,32,33]. However, this may not be a feasible or affordable solution for health care organizations because organizations tend to seek solutions that are “high-value, low-cost” [17].

The use of digital health technologies is an approach used to facilitate safe hospital-to-home transitions because they can augment provider roles and functions during transitions while attempting to minimize costs [34-36]. Many digital health technologies have been proposed to mitigate transition issues experienced by older adults and their caregivers and facilitate efficiency and coordination in the discharge process. For example, digital health interventions can be used to monitor older adults’ symptoms [37], provide educational material and discharge instructions [38,39], and facilitate timely information sharing among providers across settings [40]. However, digital health technologies, in general, have not been well integrated into clinical practice settings because of persistent barriers, including poor fit with providers’ roles and functions because digital health interventions add additional functions to the existing workloads of providers [41,42]. An improved understanding of which providers are involved in care transitions and how the technologies can support their existing provider functions may address some of these implementation barriers [43,44].

### Objectives

Despite the vast landscape of digital health technologies, there have been limited syntheses of digital health interventions used to support hospital-to-home transitions and the lessons learned from their implementation. This information is critical to avoid duplication of problematic factors that can limit the uptake of digital health technologies within the development and implementation of new transition-focused digital health interventions. To guide the development of an information communication technology to support transitions from hospital to home (the Digital Bridge intervention [45]), the specific objectives of this review were as follows:

- Understand the various roles and functions of health care providers supporting hospital-to-home transitions for older adults, allowing future technologies to be more targeted to support their work.
- Describe the types of digital health interventions used to facilitate the transition from hospital to home for older adults and elucidate how these interventions support the roles and functions of providers.
- Describe the lessons learned from the design and implementation of these interventions.
- Identify opportunities to improve the fit between technology and provider functions within the Digital Bridge intervention and other transition-focused digital health interventions.

## Methods

A rapid review methodology [46] was suitable for this review because we intended to generate a timely overview of the existing landscape of digital health technologies. This rapid review was based on our previously published protocol [43].

### Phase 1: A Selective Literature Review to Understand Roles and Functions of Health Care Providers Supporting Hospital-to-Home Transitions

A selective review [47,48] was undertaken using MEDLINE (Ovid) and Google Scholar on September 19, 2020, to provide greater insights and clarity regarding health care providers’ roles and their essential functions [44] in supporting hospital-to-home transitions. These 2 databases were selected for the following reasons: (1) they are multidisciplinary, (2) MEDLINE (Ovid) is a widely used database to identify peer-reviewed health-related literature [49], and (3) Google Scholar is a “powerful addition to other traditional search methods” to help identify known studies [50]. A selective literature review limited the search to “key studies that significantly contribute to our understanding” [47,48]. The search terms included concepts related to *navigation*, *hospital-to-home transition*, and *older adults* [43]. Any study design published in English that identified a role and function related to a hospital-to-home transition was included. The following data were extracted from relevant articles:

1. What provider role (ie, job title) is identified?
2. What is the provider’s function (ie, responsibilities related to supporting a hospital-to-home transition)?

Key roles were identified, and their functions were thematically analyzed on NVivo 11 (QSR International) using inductive thematic analysis [51]. Subsequently, the coded functions were organized according to the 3 categories of continuity of care: informational, management, and relational [52]. These categories were used because they could create a shared understanding and language for continuity of care across disciplinary and organizational boundaries [52].

### Phase 2: Identifying Digital Health Technologies Supporting Transitions

#### Literature Search

In phase 2, MEDLINE (Ovid), CINAHL (EBSCO), and Embase (Ovid) were searched on November 26, 2020, to identify literature on digital health interventions supporting the transition from hospital to home for older adults (Multimedia Appendix 1). These databases were selected because they (1) could identify health-related literature and (2) were determined by our research team (including a medical librarian HVC) to be appropriate for the scope of our search [43]. The review adhered to the PRISMA-S (Preferred Reporting Items for Systematic Reviews and Meta-Analyses extension for Literature Search) checklist [53]. The reference lists of some included articles (n=20) were hand searched, and content experts (n=6) were consulted to identify additional studies.

#### Study Selection

The search results were uploaded to the Covidence website. On the basis of the inclusion and exclusion criteria (Multimedia Appendix 2 [54,55]), each article’s title and abstract were screened by a single reviewer from the screening team (HS, TT, KK, RT, DP, MH, CMJ, AA, or JXN), followed by a full-text review conducted independently by 2 reviewers from the screening team. Any conflicts were resolved through team discussions. Studies were included if they tested a digital health intervention that supported a hospital-to-home transition for older adults and were published in or after 2010. For this review, an intervention that *supported a hospital-to-home transition* had to have recruited participants before their hospital discharge and continued in the home or community setting. The studies had to include ≥1 older adult but did not need to focus on older adults exclusively. No limits were imposed on study design to ensure that we included relevant studies, but articles had to report findings from empirical studies. Given that we intended to inform recommendations for the Digital Bridge intervention [45], a high-technology intervention for use in a “high-income country,” strictly telephone-based interventions, and interventions tested in a “low-income country” were excluded [54].

#### Data Extraction

The following data were extracted from the articles using a customized form informed by the Template for Intervention Description and Replication framework [56]: author details; country and year of publication; sampling strategy; inclusion and exclusion criteria; the medium of technology, function of technology, and who provided the intervention; study findings; and limitations and future directions. Data regarding intervention effectiveness were not extracted, reported, or synthesized in this review because this was outside its purpose, scope, and intent [57].

#### Data Analysis

We descriptively reported study characteristics and qualitatively analyzed data using a thematic analysis [51]. We first analyzed each study’s discussion using data-driven codes to identify *lessons learned*. We then coded data deductively by grouping the technology functions according to the 3 categories of continuity of care described by Haggerty et al [52]. The technology functions and providers involved in intervention delivery were compared with the provider roles and functions identified in phase 1.

## Results

### Phase 1

The literature review revealed several provider roles that commonly support hospital-to-home transitions (Textbox 1). In addition to the professional roles of allied health clinicians, pharmacists, nurses, and physicians, several navigation-specific roles were noted. Key provider functions during transitions are presented in Textbox 2. Of note, roles and functions supporting transitions differed by type of institution and many roles performed overlapping functions.

**Provider roles identified as engaged in facilitating hospital-to-home transitions.**

**Navigation-specific roles: providers with known navigation-related role titles [58]**

- Advanced practice navigator, care manager (could be a nurse, social worker or clerical staff [59]), care or program coordinator, care transition nurse, case manager, discharge coordinator, discharge liaison nurse or liaison nurse, discharge planner or facilitator or discharge planning nurse (typically a social worker or nurse [60]), case manager, discharge coordinator, geriatric care manager, guided care nurse, intensive geriatric service worker, nurse navigator, post–acute care coordinator (typically allied health or nurse [61]), patient navigator, surgical coordinated transitional care program nurse, transition coach

**Allied health**

- Occupational therapist, physiotherapist, social worker

**Pharmacist**

- Hospital or community pharmacist

**Nursing**

- Trained nurse (trained in device use), research nurse, cancer nurse specialist, telemedicine nurse, rehabilitation nurse, nurse tutor, nurse practitioner, registered nurse, chronic obstructive pulmonary disease nurse, clinical nurse specialist, community nurse, telemedicine nurse

**Physician**

- Community physician (eg, primary care physician, ambulatory physician, or community physician), hospital physician (eg, hospitalist, resident, or most responsible physician), specialist

**Key functions performed by providers during the hospital-to-home transitions.**

**Informational continuity: “The use of information on past events and personal circumstances to make current care appropriate for each individual” [52]**

- Communicate or liaise: communication or liaising with patients, caregivers, and other providers
  - Ensure the flow of information across multidisciplinary teams in the same or different sectors [62,63]. Advise and share relevant information about the patient with other providers (eg, primary care provider) [64-67]. Coordinate with other providers to ensure that services, resources, and equipment are set up for the patient. Make connections with community-based services and resources [68,69]. Communicate with patients and caregivers promptly [63]. Inform patients and caregivers and family when and how they will be contacted and whom to follow-up with if they do not receive follow-up [60,68,70,71]
- Educate: providing education to patients and caregivers
  - Educate patients about condition, disease management, symptoms, adverse events or red flags, symptom management, dietary recommendations, medication instructions, general condition or health, explain care protocols [25,68,72-81], reinforce education (eg, teach-back strategies) [82], and provide verbal or written instructions and demonstrations [63,83]
- Knowledge: providers having relevant knowledge
  - Have solid knowledge about disease and treatment, community services, where patients can seek support, and the best practices [79]. Be familiar with available community services and their eligibility
- Support or resource: providing relevant information to patients and caregivers
  - Provide informational or social support and personalized hospital-to-home support [83,84]
- Counsel (fell within 2 different categories): providing advice and recommendations to motivate behavior change
  - Provide medication, rehabilitation, dietary, or emotional counseling to patients and caregivers to motivate behavior change [79-81,85]
- Document: documenting relevant information accurately
  - Document all actions and entire plan to ensure timely information exchange between providers and ownership of the accuracy and completeness of the information [65,86]

**Management continuity: “A consistent and coherent approach to the management of a health condition that is responsive to a patient’s changing needs” [52]**

- Confirm and verify: confirming and verifying that appropriate processes and procedures were carried out to ensure continuity of care
  - Confirm that discharge summaries have complete information about a patient [87] and are sent to the team [75]. Ensure that follow-up appointments and services have been scheduled [65,75,78,88]. Confirm that patients and caregivers and families understand discharge instructions and that logistics are in place in preparation for discharge [75]. Verify that the appropriate practitioners are involved [75]
- Plan: creating a personalized care plan for patients
  - Create or contribute to a patient’s care plan based on knowledge of the patient’s individual needs and goals [89]
- Refer: referring patients and caregivers to appropriate services and resources
  - Refer patients to appropriate community services and resources to maintain continuity of care after discharge (eg, transportation) [62,72,90-93]
- Assist in navigation: helping patients and caregivers to navigate the health system
  - Assist patients in navigating through complex health systems and discharge pathways [62,72,90-93]
- Advocate: advocating patients’ access to appropriate resources and services
  - Advocate for access and entry to appropriate health and social services across settings and providers to ensure that patients’ needs are met, and break down health system and communication barriers [58,63,94]
- Follow-up: following up with patients and caregivers after discharge
  - Postdischarge follow-up and outreach with patients to identify unmet needs [95]
- Arrange or set up: facilitating access to different providers, services, and resources
  - Coordinate with different providers and services to arrange and organize timely access to postdischarge appointments and services, including primary care, medication delivery, medical devices, and transportation. Assist patients and caregivers and families in meeting their health care needs (eg, assistance completing forms) [25,58,61,62,90,91,96-98]
- Assess patients’ needs: assessing patients’ various needs to support safe transitions
  - Have a comprehensive knowledge of the patients’ care needs (eg, “patient’s medical, functional, cognitive, affective, psychosocial, nutritional, and environmental status” [76,99]) and goals to inform care and discharge plan through assessment findings [58,63,100]. Assess patients’ needs for home care and community support and resources, and identify and address potential medication adherence issues to prevent readmission [63,67,76,78,83,95,101,102]
- Direct care provision: clinical intervention
  - Provide in-person and hands-on clinical care (eg, medical, nursing, or rehabilitation intervention) [58]
- Manage: manage health and social care and needs during transitions
  - Be a manager of the patient’s care and discharge pathways [72,89]
- Monitoring: activities conducted to monitor patients’ status after discharge
  - Monitor patients for medical, health, physical, or functional status declines or the inability to self-manage their condition [86,87]. Monitor the results of medical tests and treatment adherence [89]. Conduct ongoing evaluations of the discharge plan and patient and caregiver and family needs (eg, through home visits) and create a new action plan or refer to other providers if necessary [63,89]
- Improve: improving care based on organizational quality improvement initiatives
  - Participate in quality improvement plans [59]
- Prepare: preparing providers, patients, and caregivers for patients’ discharge
  - Prepare personalized discharge plans with the patient, caregiver and family, and providers and complete discharge preparation, including determining discharge location [62,89-91]. Prepare discharge hand-over sheets [75]. Prepare a community care plan [96]

**Relational continuity: “An ongoing therapeutic relationship between a patient and one or more providers” [52]**

- Collaborate: work with patients, caregivers, and other providers to manage care
  - Collaborate with patients, caregivers and family, and other providers (eg, hospital physician-primary care physician) to create care plans [60,103]
- Empower: facilitate patient and caregivers’ involvement in the case
  - Facilitate active participation of patients and caregivers and family in care and integrate them as full partners in decisions about treatment [60,85,89,104]
- Counsel (2 categories): providing counseling to patients and caregivers in an understandable way
  - Provide individual medication counseling and ensure that patients can comprehend medication instructions and potential side effects of medication [80]. Provide emotional or dietary counseling and counseling regarding the patients’ rehabilitation needs to motivate behavior change [69,79,85]
- Coaching: providing coaching and guidance to patients and caregivers
  - Provide clinical advice, troubleshoot problems, and provide coaching about self-management skills [72,85,104-106]. Answer questions regarding concerns or issues from patients or caregivers and family [107]. Inform patients about what to expect during the transition and provide tips on communication with providers [82]
- Rapport: building relationships with patients and caregivers
  - Develop rapport and trusting relationships with patients and caregivers or family [25,98,108-110]

### Phase 2

#### Overview

**Figure 1:**
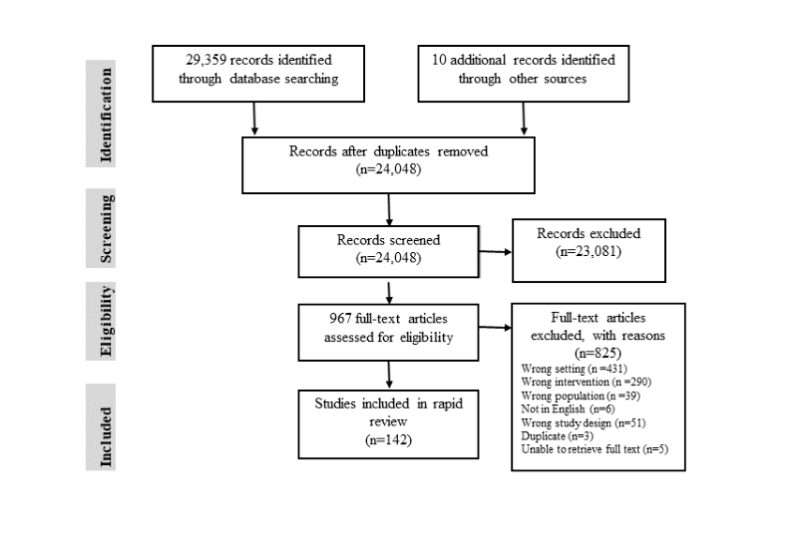
PRISMA (Preferred Reporting Items for Systematic Reviews and Meta-Analyses) flow diagram. Adapted from Moher et al [111].

The phase 2 database search identified 29,359 articles. Additional articles (n=10) were identified from hand-searching reference lists of the included articles. After removing duplicates, 81.88% (24,048/29,369) remained for the title and abstract screening and 4.02% (967/24,048) met the criteria for full-text review. Of these 967 articles, 142 (14.7%) met the study inclusion criteria (see Figure 1 [111] for the PRISMA [Preferred Reporting Items for Systematic Reviews and Meta-Analyses] flow diagram). Table 1 provides details of the study characteristics.

**Figure 2:**
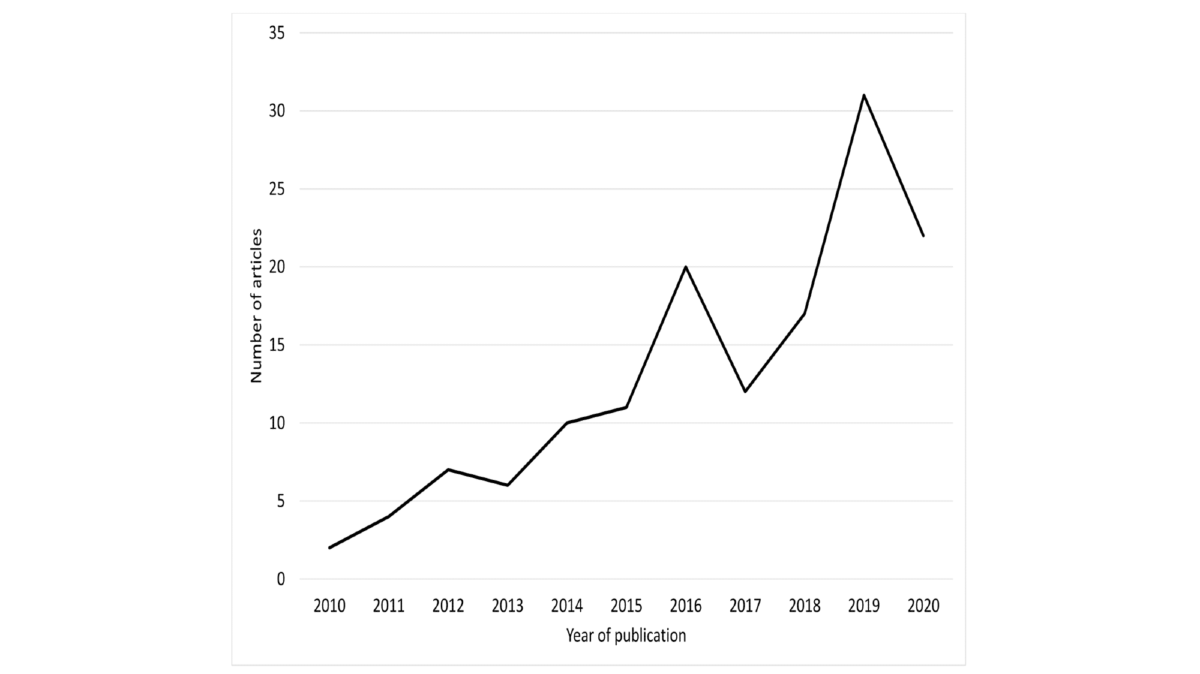
Year of article publication.

The studies were conducted in multiple countries, most of them in the United States (Table 1). They were published between 2010 and 2020, with a growing rate of publications over the years (Figure 2).

**Table 1:** Study characteristics.

Study	Country	Medical condition and intervention details
Amir et al, 2017 [112]	Israel	Cardiac (heart failure)
Ammenwerth et al, 2015 [113]	Austria	Cardiac (coronary heart disease)
Amroze et al, 2019 [114]	United States	Non–condition-specific criteria
Andikyan et al, 2012 [115]	United States	Cancer (gynecologic cancer)
Arcilla et al, 2019 [116]	United States	Multiple chronic conditions (eg, congestive heart failure, chronic obstructive pulmonary disease, and diabetes mellitus)
Austin et al, 2012 [34]	United States	Cardiac (congestive heart failure)
Avery et al, 2019 [117]	United Kingdom	Cancer (major abdominal surgery, including surgery for esophageal, gastric, or hepato-pancreato-biliary cancer)
Aziz et al, 2011 [118]	United Kingdom	Surgery (abdominal surgery)
Backman et al, 2020 [119]	Canada	Orthopedic (after hip fracture)
Barken et al, 2018 [120]	Norway	Respiratory (chronic obstructive pulmonary disease)
Barnason et al, 2019 [121]	United States	Cardiac (coronary artery bypass surgery or percutaneous coronary intervention)
Bednarski et al, 2019 [122]	United States	Cancer (colorectal cancer surgery)
Belarmino et al, 2019 [123]	United States	Cancer (radical prostatectomy)
Bernocchi et al, 2016 [124]	Italy	Neurological (stroke)
Bernocchi et al, 2012 [125]	Italy	Multiple conditions (chronic obstructive pulmonary disease, cardiac, dermatologic, diabetes, pulmonological, traumatic brain injury, and stroke)
Boeni et al, 2015 [126]	Switzerland	Diabetes
Book et al, 2013 [127]	Germany	Cancer (prostate, bladder, kidney, breast, or other types of cancer)
Bouwsma et al, 2018 [128]	Netherlands	Surgery (gynecological surgery)
Bouwsma et al, 2018 [35]	Netherlands	Surgery (gynecological surgery)
Campbell et al, 2019 [129]	United States	Orthopedic (total knee or hip arthroplasty)
Carrier et al, 2016 [130]	France	Cancer (colorectal surgery)
Chang et al, 2020 [131]	China	Cancer (esophagectomy)
Chen et al, 2010 [132]	Australia	Patients admitted to the aged care hospital ward
Chen et al, 2019 [133]	China	Cardiac (chronic heart failure)
Chiang et al, 2012 [134]	China	Cardiac (chronic heart failure)
Cox et al, 2018 [135]	United States	Medical and surgical intensive care unit patients (receipt of mechanical ventilation for >48 consecutive hours and successful extubation before discharge)
Cox et al, 2019 [136]	United States	Cardiac (cardiorespiratory failure)
Davis et al, 2015 [137]	United States	Multiple conditions (acute chronic disease)
Day et al, 2018 [138]	United States	Orthopedic (total joint arthroplasty)
Dendale et al, 2012 [139]	United States	Cardiac (severe heart failure)
DeVito Dabbs et al, 2016 [140]	United States	Surgery (lung transplantation)
DeVon et al, 2010 [141]	United States	Cardiac (coronary heart disease)
Dexter et al, 2013 [142]	United States	Orthopedic (total hip replacement)
Dorothy et al, 2016 [143]	United States	Cardiac (cardiovascular surgery)
Duncan et al, 2018 [144]	United States	Neurological (stroke and transient ischemic attack)
Dunn et al, 2015 [145]	United States	Patients on medical or surgical units on warfarin
El-Kareh et al, 2012 [44]	United States	Patients with positive and untreated or undertreated blood, urine, sputum, or cerebral spinal fluid cultures
Evangelista et al, 2015 [146]	United States	Cardiac (chronic heart failure)
Finn et al, 2011 [13]	United States	Patients on medical service
Fitzsimmons et al, 2016 [147]	United Kingdom	Respiratory (chronic obstructive pulmonary disease)
Frail et al, 2016 [148]	United States	Patients taking ≥1 long-term medication
Gesell et al, 2019 [149]	United States	Neurological (stroke)
Gunter et al, 2018 [150]	United States	Surgery (vascular surgery)
Gurwitz et al, 2014 [40]	United States	Patients being discharged from an inpatient unit
Gustavell et al, 2019 [151]	Sweden	Cancer (pancreaticoduodenectomy)
Gustavell et al, 2019 [152]	Sweden	Cancer (pancreaticoduodenectomy)
Haynes et al, 2020 [153]	United States	Cardiac (decompensated heart failure)
Heaton et al, 2019 [154]	United States	Multiple conditions (acute myocardial infarction, pneumonia, congestive heart failure, chronic obstructive pulmonary disease, or diabetes)
Heiney et al, 2020 [155]	United States	Cardiac (heart failure)
Hewner et al, 2014 [156]	United States	Multiple conditions
Ho et al, 2016 [157]	China	Respiratory (chronic obstructive pulmonary disease)
Holleck et al, 2017 [158]	United States	Patients admitted to medical service
Holt et al, 2011 [159]	United States	Surgery (plastic surgery)
Hu et al, 2014 [160]	China	Cardiac (percutaneous coronary intervention)
Jayaram et al, 2017 [161]	United States	Cardiac (heart failure)
Jeungok et al, 2017 [162]	United States	Orthopedic
Jonker et al, 2020 [163]	Netherlands	Cancer (elective oncologic resection of a solid tumor)
Kamoen et al, 2020 [164]	Belgium	Neurological (ischemic stroke)
Kang et al, 2019 [165]	China	Neurological (stroke)
Karapinar-Çarkit et al, 2014 [166]	Netherlands	Patients discharged from the cardiology and respiratory wards
Katz et al, 2016 [167]	United States	Cancer (pancreatectomy)
Keeping-Burke et al, 2013 [168]	Canada	Cardiac (coronary artery bypass graft surgery)
Khan et al, 2018 [169]	Denmark	Cardiac (on- or off-pump coronary artery bypass graft or heart valve surgery)
Klement et al, 2019 [170]	United States	Orthopedic (total joint arthroplasty)
Kogut et al, 2014 [171]	United States	Chronic medical conditions
Lacson et al, 2018 [172]	United States	Respiratory (pulmonary nodules)
Lafaro et al, 2020 [37]	United States	Cancer (colorectal, gastric, pancreatic, and liver cancer surgery)
Lavu et al, 2019 [36]	United States	Surgery (pancreaticoduodenectomy)
Layton et al, 2014 [173]	United States	Cardiac (coronary artery disease or congestive heart failure)
Lehnbom et al, 2014 [174]	Australia	Patients discharged from a hospital unit
Lin et al, 2020 [175]	China	Cardiac (coronary artery disease)
Lindhardt et al, 2017 [176]	Denmark	Patients admitted to internal medicine units and at nutritional risk
Lowres et al, 2016 [177]	Australia	Cardiac (cardiac surgery)
Luo et al, 2019 [178]	China	Orthopedic (total hip arthroplasty)
Lyu et al, 2016 [179]	China	Cancer (head and neck tumor)
Madigan et al, 2013 [180]	United States	Cardiac (heart failure)
Markle-Reid et al, 2020 [181]	Canada	Neurological (stroke and multimorbidity)
Martirosov et al, 2020 [182]	United States	Patients admitted to hospital
Mathar et al, 2015 [183]	Denmark	Respiratory (chronic obstructive pulmonary disease)
McCloskey et al, 2015 [184]	Canada	Patients discharged from geriatric rehabilitation
McGillion et al, 2020 [185]	Canada and United Kingdom	Cardiac and major vascular surgery
Melholt et al, 2018 [186]	Denmark	Cardiac (ischemic heart disease or heart failure, including patients who had undergone coronary artery bypass or valve surgery)
Meng-Yao et al, 2020 [187]	China	Neurological (stroke)
Metcalf et al, 2019 [188]	United States	Cancer (radical cystectomy)
Moffet et al, 2015 [189]	Canada	Orthopedic (total knee arthroplasty)
Moro Agud et al, 2016 [190]	Spain	Patients admitted to a hospital unit
Mousa et al, 2019 [191]	United States	Surgery (arterial revascularization with groin incision)
Moy et al, 2014 [192]	United States	Patients admitted to medical service
Nazar et al, 2016 [193]	United Kingdom	Patients on ≥4 medicines or had changes in medicines during the hospital stay
Newnham et al, 2015 [194]	Australia	Patients discharged from the acute general medical ward
Nielsen et al, 2020 [195]	Denmark	Surgery (kidney transplantation)
Nilsson et al, 2020 [196]	Sweden	Cancer (prostate cancer surgery)
Nundy et al, 2013 [197]	United States	Cardiac (heart failure)
Ong et al, 2016 [198]	United States	Cardiac (heart failure)
Ostrovsky et al, 2016 [199]	United States	Non–condition-specific criteria (medical fee-for-service patients)
Park et al, 2017 [200]	South Korea	Orthopedic (total knee replacement)
Pastora-Bernal et al, 2018 [201]	Spain	Orthopedic (arthroscopic subacromial decompression)
Pavic et al, 2020 [202]	Switzerland	Cancer (palliative cancer care)
Pavic et al, 2020 [203]	Switzerland	Cancer (palliative cancer care)
Pedone et al, 2015 [204]	Italy	Cardiac (heart failure)
Piau et al, 2019 [205]	United States	Cancer
Piette et al, 2020 [206]	United States	Patients admitted with an illness that is associated with increased rehospitalization risk
Ponce et al, 2016 [207]	United States	Surgery (neurosurgical or orthopedic)
Prince et al, 2019 [208]	United States	Cancer (hematologic malignancies)
Ramkumar et al, 2019 [209]	United States	Orthopedic (total knee arthroplasty)
Reed et al, 2020 [210]	United States	Diabetes
Reider-Demer et al, 2018 [211]	United States	Neurological (elective neurosurgery)
Requena et al, 2019 [212]	Spain	Neurological (stroke)
Ritchie et al, 2016 [213]	United States	Multiple conditions (heart failure and chronic obstructive pulmonary disease)
Sabir et al, 2019 [214]	United Kingdom	Non–condition-specific criteria
Saleh et al, 2014 [215]	Norway	Respiratory (chronic obstructive pulmonary disease)
Santana et al, 2017 [216]	Canada	Patients admitted to medical teaching units with multiple comorbidities and complicated medication profiles
Scheper et al, 2019 [217]	Netherlands	Orthopedic (joint arthroplasty)
Schneider et al, 2017 [218]	United States	Neurological (stroke)
Sinha et al, 2019 [219]	United States	Patients admitted to general medicine service
Smith et al, 2016 [220]	United States	Patients admitted to general medicine, geriatrics, or cardiology inpatient services; medically complex (≥2 comorbid conditions)
Sorknaes et al, 2011 [221]	Denmark	Respiratory (chronic obstructive pulmonary disease)
Sorknaes et al, 2013 [222]	Denmark	Respiratory (chronic obstructive pulmonary disease)
Sui et al, 2020 [223]	China	Cancer (surgical resection for non–small cell lung cancer)
Sun et al, 2017 [224]	United States	Cancer (major abdominal cancer surgery)
Sun et al, 2017 [225]	United States	Cancer (lung cancer surgery)
Tamblyn et al, 2019 [226]	Canada	Patients admitted to medical and surgical hospital units
Tamblyn et al, 2018 [227]	Canada	Patients admitted to medical and surgical hospital units
Timmers et al, 2019 [228]	Netherlands	Orthopedic (total knee replacement)
Treskes et al, 2020 [229]	Netherlands	Cardiac (myocardial infarction)
van den Berg et al, 2016 [230]	Australia	Neurological (stroke)
Van der Meij et al, 2018 [231]	Netherlands	Surgery (intermediate-grade abdominal surgery)
Vest et al, 2015 [232]	United States	Non–condition-specific criteria
Vesterby et al, 2017 [233]	Denmark	Orthopedic (fast-track hip replacement)
Vianello et al, 2016 [234]	Italy	Respiratory (chronic obstructive pulmonary disease)
Villani et al, 2014 [235]	Italy	Cardiac (heart failure)
Wade et al, 2012 [236]	Australia	Frail older adults with multiple chronic conditions
Wang et al, 2017 [237]	China	Respiratory (chronic obstructive pulmonary disease)
Wang et al, 2018 [238]	China	Cancer (colorectal cancer or other digestive and urinary tumors and permanent stoma after surgery)
Wang et al, 2018 [239]	China	Orthopedic (hip replacement surgery)
Wan et al, 2018 [240]	China	Neurological (hypertensive ischemic stroke)
Whitehouse et al, 2020 [241]	United States	Diabetes
Wilcock et al, 2019 [242]	United Kingdom	Patients admitted to a hospital
Wolf et al, 2016 [38]	Sweden	Cardiac (acute coronary syndrome)
Zheng et al, 2019 [243]	China	Orthopedic (total joint arthroplasty)
Zhou et al, 2019 [244]	China	Cancer (breast cancer surgery)
Zhou et al, 2020 [245]	China	Cancer (breast cancer surgery)

#### Participants Targeted

##### Medical Conditions and Interventions Targeted

Digital health interventions were most frequently used to facilitate transitions for cardiac conditions (eg, cardiac surgery and chronic heart failure; 28/142, 19.7%) and cancer (eg, cancer surgery and cancer management; 26/142, 18.3%). Fewer digital health interventions targeted patients admitted to specific hospital units (eg, geriatric, medical, or intensive care unit; 19/142, 13.4%) and patients with multiple conditions (12/142, 8.5%), orthopedic conditions (16/142, 11.3%), neurological conditions (eg, stroke and brain tumor; 12/142, 8.5%), other surgical interventions (eg, after kidney transplantation; 11/142, 7.7%), and respiratory conditions (eg, chronic obstructive pulmonary disease management; 10/142, 7%). In addition, a small number of digital health interventions supported transitions for patients who had diabetes (3/142, 2.1%) or non–condition-specific criteria (eg, age group and medical health plan; 5/142, 3.5%).

##### Age Groups Targeted

In total, 15.5% (22/142) of the included interventions were conducted with samples of strictly older adults. Other interventions did not specify a targeted age range within their inclusion criteria (54/142, 38%) or had included participants aged 18 to 21 years or older (49/142, 34.5%).

### Details of Digital Health Technologies

#### Intervention Type

Of the 142 interventions, 47 (33%) were classified into multiple categories of intervention types (N=193 intervention classifications). Of the 6 intervention-type characterizations, smartphone, tablet, or web-based interventions (91/193, 47.2%) were the most common than telemonitoring and wearables, clinical documentation system (45/193, 23.2%), clinical documentation systems (29/193, 15%), automated telephone calls or automated SMS text messaging (14/193, 7.3%), email interventions (10/193, 5.2%) or other interventions (eg, television video; 4/193, 2.1%).

#### Provider Roles and Functions Involved in the Intervention

As shown in Table 2, a total of 35.9% (51/142) of the interventions used multiple provider roles (n=202 provider roles identified) in the implementation of the digital health intervention, with nurses (64/202, 31.7%) and physicians (61/202, 30.2%) being the most common providers of digital health interventions. Discharge-specific personnel such as a transition coach, nurse care transition coordinator, discharge facilitator, advanced practice nurse, and systems navigator were less common (18/202, 8.9%).

Some interventions had designated a study-specific health care provider to carry out the digital health intervention activities, whereas others added the responsibility onto a provider’s existing workload. The responsibilities of providers also differed based on the type and purpose of technology and whether communication between patients and providers was initiated by patient or provider. Among some interventions with patient-initiated communication, providers had to always be available for consultation during the intervention period.

The digital health interventions were most commonly used up to 7 days after discharge (29/142, 20.4%) or between 31 and 90 days after discharge (39/142, 27.5%). It was less common for the interventions to continue for 91 days to <6 months after discharge (18/142, 12.7%) or beyond 6 months after discharge (7/142, 4.9%).

**Table 2:** Provider roles and examples of involvement in technology intervention used to facilitate hospital-to-home transitions (N=202).

Provider role; providers, n (%)	Specific examples	Examples of provider role–technology interactions
Physician; 61 (30.2)	Community physician (eg, primary care physician, ambulatory physician, and community physician), hospital physician (eg, hospitalist, resident, and most responsible physician), and specialist (eg, cardiologist, surgeon, occupational physician, geriatrician, and pulmonologist)	Family physicians were alerted when patient data (eg, biometric or symptoms) fell outside predefined parameters and asked to visit or contact the patient [139]
Nurse; 64 (31.7)	Specially trained nurse (trained in device use), research nurse, cancer nurse specialist, telemedicine nurse, rehabilitation nurse, nurse tutor, nurse practitioner, registered nurse, chronic obstructive pulmonary disease nurse, clinical nurse specialist, and community nurse	They reviewed all transmitted biometric and symptom data, flagged patients whose data fell outside the predefined parameters, and communicated with or assessed patients using communication technology [153,168]
Clinician; 19 (9.4)	Discipline not specified	Clinicians were alerted when patient responses were outside predefined parameters, and they reviewed flagged responses [161]
Allied health; 19 (9.4)	Occupational therapist, physiotherapist, social worker, and psychologist	Conducted telehealth consultations or sessions [37,183]
Pharmacist; 18 (8.9)	Hospital or community pharmacist	Access information from other providers in the same facility or across facilities, settings or receive information from them and send information to them [214]
Navigation-specific roles; 18 (8.9)	Advanced practice nurse or provider, care manager, care or program coordinator, care transition nurse, case manager, discharge planner or facilitator or discharge planning nurse, nurse navigator, post–acute care coordinator, system navigator, and transition coach	Provided 24-hour consultation, which was accessible to patients through technology [175]
Other; 3 (1.5)	Physician’s assistant, unit supervisor, surgical team’s physician’s assistant	Used to communicate with other providers and send and receive information [208]

#### Technology Functions

In terms of the technology functions that supported hospital-to-home transitions, most (116/142, 81.7%) of the technologies fell into multiple categories (ie, 57/142, 40.1%, fell into 2 categories and 59/142, 41.5%, fell into 3 categories).

Of the 317 total technology functions within the included interventions, 142 (44.8%) were related to *management continuity*, including following up, assessing, and monitoring patients’ status after hospital discharge, as well as facilitating referrals. Some technologies could identify values outside a predefined range during follow-up, assessment, and monitoring of patients’ status. However, others required human resources to review all data to identify abnormal values. In both cases, if values fell outside the range, a human resource (eg, provider or study personnel) had to follow-up and provide appropriate guidance and immediate treatment or the technology instructed a patient to initiate contact with a provider. *Informational continuity* was supported among 32.2% (102/317) of the identified technology functions, including facilitating communication (eg, between inpatient and outpatient providers or between patient and provider) and educating patients and caregivers. *Relational continuity* (eg, counseling and rapport building) was least supported by the technologies (73/317, 23%).

#### Outcomes of Interest

Of the total outcomes of interest (n=315) examined in the articles, more than half of the outcomes evaluated the effect of the intervention on patient-level factors (eg, disease knowledge, quality of life, and changes in physical or psychological functioning) and technology-user interactions (eg, use of technology, patient satisfaction with technology, and the perceived value of technology) at 28.6% (90/315) and 28.3% (89/315), respectively. Of all outcomes, 17.5% (55/315) related to health care use, examined through health care–related costs and hospital readmission rates or emergency department visits at various time points (eg, 30, 60, 90, and 180 days after discharge). The intervention effect on provider-related outcomes (eg, changes in provider workflows, provider burden, and clinical documentation accuracy), implementation-related outcomes (eg, compliance; 9/315, 2.9%), and caregiver- and family-related outcomes (eg, caregiver stress; 3/315, 1%) were less commonly examined (23/315, 7.3%). *Other* outcomes (eg, documentation time, economic evaluations; 46/315, 14.6%) were measured.

### Lessons Learned From Digital Health Interventions

The lessons learned from the interventions pertained to challenges (eg, researcher-identified limitations or challenges of interventions) and opportunities (eg, researcher-identified strengths of interventions and recommendations); these were categorized into two broad categories: (1) technology-related and (2) research process–related (Table 3).

**Table 3:** Summary of the lessons learned from implementation of digital health interventions.

Challenge and description				Examples
**Technology-related challenges**				
	**Usability issues**			
		Participants’ physical, functional, and sensory function	Low vision Hand tremor	
		Patients’ and providers’ lack of technical skills and experience	Forgetting log-in information or not remembering to charge the device Accidentally disabling device features Low technology comfort	
		Device-related technical issues	Internet connectivity issues Software updates affecting function Immaturity of the prototype	
		Fit and compatibility issues	Poor fit with patients’ or providers’ routine Device incompatible with older devices Not integrated into organization’s electronic documentation system Identifying provider functions rather than their roles may enable the technology to accommodate differences among jurisdictions and changing scopes of practice	
	**Technology content and function**			
		Patient-facing content	Hypertext links were distracting and confusing Language too technical Offensive tone and complexity of the wording Symptom-reporting questions too specific or broad caused misunderstanding	
		Expectations of patient-initiated provider contact	Not all participants were confident about the appropriate circumstances in which to contact the provider	
		Device notifications	Excessive alerts caused “alert fatigue” and resulted in less attention being paid to the alert or ignoring it altogether	
**Technology-related opportunities**				
	**Technology function and features**			
		Enhancing functionality	Address and improve multiple components of the transition process	
		Accessibility, adaptations, and customization	Low-vision adaptations Adapt for participants with low technological literacy and no social support Self-directed apps Use of personal devices when possible and compatibility across multiple data and operating systems Provision of the device when participants do not have access to a personal device	
		Training	Technical setup Training on technology use Engage caregivers in the intervention when possible	
		Fit with workflows, workloads, and buy-in	Participants, family, caregivers, and providers should inform the technology design and how technology could be integrated into the day-to-day practices of all stakeholders Accounting for providers’ ethical, legal, and professional responsibilities	
**Research process–related challenges**				
	**Data collection**			
		Recruitment and retention challenges	Lack of interest High attrition	
		Small sample size	Unable to explore the relationship between participants’ profiles, participants’ adherence and compliance to intervention or conduct subgroup analyses	
		Sampling bias	Homogenous samples Inclusion limited to those with technology comfort or access	
		Missing data	Impacting reliability of intervention results	
		Outcome measures	Outcome measures such as rehospitalization and survival may not be sufficiently sensitive to determine intervention impact Single-blinded evaluator could introduce measurement error	
		Interventions across settings or institutions	Cross-setting coordination challenges	
**Research process–related opportunities**				
	**Data collection**			
		Recruitment considerations	Video of 10-to-15–minute duration describing the intervention (potential benefits and utility) during recruitment to reduce apprehension Consideration of low compliance rates within sample size calculations Comparing the characteristics of participants with those of individuals who declined can indicate selection bias and affect the intervention’s generalizability and acceptability	
		Outcomes	Careful consideration of outcome measures (eg, objective or subjective) and end points	
		Missing data	Begin intervention during hospitalization Schedule follow-ups during routine patient visits to minimize data lost during follow-up	

### Technology-Related Challenges and Opportunities

Technology-related challenges and opportunities pertained to the use of the devices.

#### Technology-Related Challenges

Among digital health interventions, researchers reported usability issues with the technology because of patients’ physical condition [177], patients’ or providers’ lack of technical skills and experience [159,176,177,202], and the technology not fitting into patients’ or providers’ routine and workflow [119,173]. Regarding fit with workflows, researchers emphasized that health care providers operate within regulated environments; ethical, legal, and professional considerations related to providers’ roles and care settings had to be accounted for in the design of digital health technology interventions [148,159,227]. Technical issues such as internet connectivity issues, software updates, or immaturity of the prototype [148,152,177] also decreased usability and interfered with the technology’s function (eg, restricted data transfer and alert failure) [152,202]. In addition, some researchers encountered compatibility issues with older devices and other organizations’ electronic documentation systems, which created usability issues [44,145,148,150,192,210,212].

In terms of the technical content, researchers found that some participants perceived the patient-facing content as problematic because of the technical language, tone, or complexity [152,162], as well as hypertext links that caused confusion [39,152]. Patient-initiated technology functions also presented a challenge because not all participants could use the functions or follow the instructions as intended [196]. Researchers also found it challenging to set alerts that would be appropriate for all patients because excessive alerts caused “alert fatigue” and resulted in less attention being paid to the alert or ignoring it altogether [40,44,152,157].

#### Technology-Related Opportunities

A few researchers emphasized that designing digital health interventions to address and improve multiple components of the transition process may enhance functionality [119,145]. In addition, they indicated that technology accessibility, adaptations, or customization could accommodate individual preferences and increase applicability to different populations [39,140,205,227]. Researchers indicated that increasing accessibility could start with providing the technology to participants without a personal device to reduce disparities of access based on technology ownership [150]. Researchers suggested using participants’ devices to enhance usability when possible, which may require compatibility across multiple data and operating systems [122,167,212]. In addition, technical setup and training on using the technology and engaging caregivers in the intervention could support the usability and intervention quality, safety, and adherence [112,135,157]. Moreover, building self-directed functions might help overcome logistical barriers associated with scheduled interventions [136]. This finding extends to timely feedback because researchers found that participants wanted to be notified when providers had reviewed their responses [150].

To address the technology’s fit with workflows, workloads, and buy-in, some researchers believed that participants, family, caregivers, and providers should be engaged in helping to design the intervention [136,148,228]. Researchers found that interventions that placed high accountability and responsibility on health care providers and added additional work to their workload resulted in provider-related usability issues because providers “struggled to find time in their day” to carry out intervention activities [40,150,195]. Researchers noted that identifying functions rather than provider roles may enable the technology to accommodate differences among jurisdictions (eg, country and institution) and changing scopes of practice over time [227]. Alternatively, if human resources are limited, interventions using automated telephone calls or central monitoring centers for multiple institutions could be considered to reduce the number of personnel and time required for monitoring [188]. Thus, understanding how technology could be integrated into the day-to-day practices of all stakeholders was an essential task for technology developers, along with helping providers envision ways to implement the technology in practice [119,181].

### Research Process–Related Challenges and Opportunities

Challenges and opportunities within the reported research processes pertained to the recruitment process, data collection, and study and intervention designs.

#### Research Process–Related Challenges

Recruitment challenges and high attrition were commonly reported within the studies [135,246]. As several interventions had a small sample size, researchers acknowledged limitations, including being unable to explore the relationship between participants’ profiles and adherence and compliance information or conduct subgroup analyses [112,135,157,205]. Researchers reported that sampling bias could have had an impact on the generalizability of their results because the samples were small [115,141,165] and homogenous (ie, primarily White) [150] and could have been exacerbated because inclusion was limited to participants with internet-enabled devices [201]. Missing data was another concern reported by researchers that may have affected the reliability of the intervention results [158].

Beyond data collection, researchers reported that interventions conducted at a single site may have reduced generalizability to other settings [160,181,218,226,241]. The study by DeVito Dabbs et al [140] indicated that outcome measures such as rehospitalization and survival may not be sufficiently sensitive to identify the impact of a technology intervention.

Researchers found that effectively integrating the technology in clinical environments would likely require early engagement with patients and providers, support from senior leadership, integration within existing electronic systems [119,144,148,166], and testing of technologies in real-world settings to identify implementation barriers [140]. Finally, researchers of a digital health technology intervention that operated across settings or institutions reported challenges with coordination among providers in hospital, primary care, and community settings [148].

#### Research Process–Related Opportunities

Several researchers recommended more extensive and diverse participant samples in future digital health interventions [150,171,173,247] and consideration of low compliance rates within sample size calculations [173]. They believed that providing participants with an explanation of the potential benefits and utility of the technology may also enhance study participation [173]. In addition, comparing the characteristics of participants with those of individuals who declined participation gave researchers insight into selection bias and the intervention’s generalizability and acceptability [151,169,231,237].

Regarding outcomes of interest, researchers advised carefully considering which outcome measures (eg, objective or subjective) [173,224] and end points to use [186,193]; multicenter studies with longer follow-up time (ie, >30 days after discharge) might be required to observe the intervention’s effect on patient-clinician relationships [160,181]. Opportunities identified by researchers to improve data include analyzing technology log data for objective data on patients’ and providers’ use of technology [186,193], beginning the intervention within the hospital setting, and incorporating the follow-ups into routine patient visits to potentially minimize data lost during follow-up [141,148].

## Discussion

### Recommendations

This rapid review provides an overview of digital health interventions supporting hospital-to-home transitions and describes how the technologies have been used to support the roles and functions of health care providers in supporting these transitions. Consistent with the aim of a rapid review approach, we have compiled a set of recommendations (Table 4) to guide the design of new and existing digital health interventions such as Digital Bridge that support hospital-to-home transitions based upon the reviewed literature. Our review extends and complements the existing literature [41,42,248] by highlighting transition-specific considerations within the design and implementation of future digital health interventions that better support provider roles and functions during transitions.

**Table 4:** Recommendations to guide the design and implementation of digital health interventions to facilitate hospital-to-home transitions.

Recommendation	Description
Recommendation 1: align the design and delivery of digital health interventions to provider functions	As roles and functions can differ based on several factors (eg, the organizations, jurisdiction, and care settings), technology functions should consider the roles and functions relevant to their target setting; alternatively, to increase generalizability, technology may need to support specific provider functions (ie, provider responsibilities) rather than outlining specific roles (ie, provider titles) Address multiple functions within transitional care, including functions supporting informational, management, and relational continuity of care Integration of technology with multiple organizations and across care settings Added provider functions with technology use should be minimal (eg, automated and self-directed functions could be integrated into interventions to reduce provider functions) Share functions related to technology use with patients and caregivers when possible Begin before or immediately after hospital admission and extend care into the community
Recommendation 2: design for, and test with, older adults	To ensure that technology functions effectively meet the transitional care needs of older adults, digital health interventions should be designed for, and tested with, older adults Consider strategies to recruit and retain older adults with poor health Consider how technology functions may affect inequities Include caregivers, when possible, in digital health interventions because they play valuable roles in hospital-to-home transitions
Recommendation 3: examine multilevel outcomes	Examine reasons for declining and dropping out of interventions Examine multilevel outcomes Provider-level outcomes may give insight into whether technology functions are perceived to support provider functions effectively Evaluate specific technology functions

#### Recommendation 1: Align the Design and Delivery of Digital Health Interventions With Provider Functions

This review demonstrates that many existing technologies that support hospital-to-home transitions encounter implementation-related barriers. The health care system is complex, and the discharge process is often “busy, rushed and emotional” [249]. During hospital-to-home transitions, patients move from one setting to another and provider functions and responsibilities become unclear because communication often fails to cross boundaries [250]. Thus, a critical lesson from this review is that digital health interventions should emphasize the provider functions that the technology supports rather than focusing on how professional groups can use solutions because roles and functions can differ by organization and care setting.

We have highlighted that many providers involved in transitions tend to have overlapping functions. We have outlined specific provider functions that could be built into the design of digital health interventions to support transitional care workflows and potentially reduce provider burden. These functions may address the factors presently limiting uptake of digital health interventions, including poor fit with providers’ functions and provider perceptions of low degree of usefulness [41,42,248]. To meaningfully support hospital-to-home transitions, digital health interventions may need to address multiple functions involved in patient care beyond primarily supporting functions related to management continuity (eg, monitoring) and informational continuity. On the basis of the findings from this review, technology functions related to relational continuity warrant further exploration. These are the components that are appropriate for technology to address and support and the ones that rely on the interface between people and technology.

Moreover, technologies should be designed to minimize the burden on providers and be designed in such a way that they can support provider functions. Although technologies demonstrate their ability to support specific provider functions such as remote monitoring and patient education, they add functions and place high levels of accountability on single providers. For instance, remote monitoring technologies could yield large quantities of data that providers then become responsible for sorting through and acting on, adding another function to their workload [120,153]. Integration of such technologies in clinical practice could be unfeasible because the added provider functions are among the prominent barriers to the uptake of technologies [41].

Perceived usefulness may be improved by highlighting how the purpose and function of the technology fit with the functions of providers during hospital-to-home transitions and whether it could result in time savings and the workload reduction of providers and by outlining the responsibilities of providers in the delivery of digital health interventions [140]. Furthermore, as technologies integrate more advanced and automated functions, the burden on providers may be reduced. For instance, automated reminders may reduce demands on providers [34]. However, advanced technologies may not be suitable for all patients and these individuals may require training to recognize red flags and when to re-engage with providers [152]. Sharing responsibility with, and facilitating more active involvement of, patients and caregivers (when appropriate) or adding trained volunteers may be another way to reduce the added responsibility faced by health care providers [251].

#### Recommendation 2: Design for, and Test Digital Health Interventions Specifically With, Older Adults

Older adults have unique transitional care needs that the providers strive to meet through their functions. Provider functions to achieve relational and informational continuity of care have been deemed necessary to achieve high-quality hospital-to-home transitions for older adults [22,252]. However, we identified these functions to be a gap in the existing digital health interventions supporting hospital-to-home transitions because these functions were least supported by technology. We believe that these should be integrated within technology functions of future digital health interventions.

Of note, this review revealed that digital health interventions were rarely designed to meet the unique needs of older adults or exclusively tested with older adults. Thus, we contend that future technology functions should be designed to meet these specific transitional care needs while also accounting for design considerations related to older adults’ complex needs, including physical, cognitive, and sensory needs [253-255]. Moreover, new strategies may be needed to recruit and retain older adults with poor health status. Using human-centered design principles, including co-designing and testing with clinicians and older adults with complex care needs, may enhance the use and effectiveness of interventions [41,248] and could reveal how better to integrate relational management into the technology functions. Furthermore, critical investigations of how the functions of existing digital health interventions may have contributed to the exacerbation of inequities are necessary to highlight new insights and guidance for functions of future interventions to eliminate such disparities [255,256].

#### Recommendation 3: Examine Multilevel Outcomes

We recommend that those leading digital health interventions examine outcomes of interest at multiple levels, including the patient, provider, organization, and system levels. Most transitional interventions examined the impact of digital health interventions on patient-level outcomes. However, not all studies had examined why participants declined or dropped out of digital health interventions, which would have provided valuable insights for future work. Provider-, organization-, and system-level outcomes were less common but are essential to consider. Although patient-level outcomes are helpful, costs and benefits need to be assessed for health care organizations and health systems, including economic feasibility and quality measures [257]. In particular, exploring patient-level outcomes can provide insight into whether the technology functions effectively support the provider functions.

Moreover, evaluating specific technology functions may provide insights into which ones may need to be refined. Researchers may also further explore the feasibility and benefits of transition-specific roles to support digitally enabled transitions because these studies were limited. In addition, reporting research-level outcomes, including insights and reflections from the research teams, may contribute valuable knowledge that could guide future interventions.

### Limitations

Several factors limit this review. First, the rapid review methodology (eg, single-reviewer title and abstract screening and limited number of databases searched) may have led to missing relevant articles. Title and abstract screening were initiated after a minimum interrater reliability among screeners of κ=0.80 (ie, sufficient interrater reliability) was achieved to reduce the risk of missing relevant articles [258]. Second, there is a lack of standardized terminology and definitions for hospital-to-home transitions, provider roles and functions, transitional interventions, and digital health technologies. Thus, our inclusion criteria were difficult to apply and we had to create additional parameters to judge whether the studies related to these areas. For example, to be considered a *hospital-to-home transition intervention*, the intervention had to begin (ie, recruitment) at the hospital and extend to the community. It is also possible that some articles that failed to provide a detailed methodology could have been mistakenly excluded. However, this review was not intended to map the relevant literature entirely but rather to provide an overview of the landscape. Third, although we planned to conduct a quality appraisal using the Mixed Methods Appraisal Tool [259,260], we decided against a formal quality appraisal for two reasons: (1) the studies did not report sufficient details of their intervention design and methods for the team to appraise their quality confidently and accurately (eg, *Is randomization appropriately performed?* and *Are outcome assessors blinded to the intervention provided?*) and (2) this review intended to focus on critical lessons learned from the processes involved in designing, delivering, and evaluating the interventions rather than the interventions’ effectiveness (eg, outcomes); thus, an appraisal was not critical to meet these objectives. We recommend that future digital health interventions report comprehensive details of their methods to enable future reviews to critically appraise them. Fourth, the inclusion criteria were modified to capture the most relevant literature and data during the review process. However, this led to deviations from the protocol (eg, excluding telephone-based interventions). Fifth, the purpose of phase 1 was to characterize typical roles and critical functions involved in transitions to create a general understanding of the context rather than to create an exhaustive list of all roles. However, we acknowledge that several roles, including the roles and functions of specialized health professionals, may not have been reflected in the results. In addition, roles and functions may also differ by factors such as the institution, country or region, and clinical setting. Thus, technology designers should consult with their intended users to ensure that the technology aligns with their roles and functions. Sixth, each article was reported as a single intervention because we could not link articles that reported a single intervention’s outcomes within multiple articles. Finally, the findings are not limited to older adults because we included any study that included at least one older adult. Nonetheless, this review provides valuable information to guide the design and implementation of existing and new digital health interventions such as the Digital Bridge.

### Conclusions

In conclusion, this review provides an overview of the landscape of digital health interventions that support hospital-to-home transitions and identifies recommendations for future studies based on the lessons learned. The findings from this review will serve as a valuable guide for the design and implementation of Digital Bridge and other digital health interventions to support hospital-to-home transitions.

## Appendix

Multimedia Appendix 1 Search strategies.

Multimedia Appendix 2 Phase 2 inclusion criteria.

## Abbreviations

PRISMA: Preferred Reporting Items for Systematic Reviews and Meta-Analyses
PRISMA-S: Preferred Reporting Items for Systematic Reviews and Meta-Analyses extension for Literature Search

## References

[1] Werner NE, Tong M, Borkenhagen A, Holden RJ (2019). Performance-shaping factors affecting older adults' hospital-to-home transition success: a systems approach. Gerontologist.

[2] Neiterman E, Wodchis WP, Bourgeault IL (2015). Experiences of older adults in transition from hospital to community. Can J Aging.

[3] Naylor MD (2004). Transitional care for older adults: a cost-effective model. LDI Issue Brief.

[4] Krumholz HM (2013). Post-hospital syndrome--an acquired, transient condition of generalized risk. N Engl J Med.

[5] Foust JB, Naylor MD, Bixby MB, Ratcliffe SJ (2012). Medication problems occurring at hospital discharge among older adults with heart failure. Res Gerontol Nurs.

[6] Andreasen J, Lund H, Aadahl M, Sørensen EE (2015). The experience of daily life of acutely admitted frail elderly patients one week after discharge from the hospital. Int J Qual Stud Health Well-being.

[7] Jencks SF, Williams MV, Coleman EA (2009). Rehospitalizations among patients in the Medicare fee-for-service program. N Engl J Med.

[8] Mesquita ET, Cruz LN, Mariano BM, Jorge AJ (2015). Post-hospital syndrome: a new challenge in cardiovascular practice. Arq Bras Cardiol.

[9] van Seben R, Reichardt LA, Essink DR, van Munster BC, Bosch JA, Buurman BM (2019). "I feel worn out, as if i neglected myself": older patients' perspectives on post-hospital symptoms after acute hospitalization. Gerontologist.

[10] Hestevik CH, Molin M, Debesay J, Bergland A, Bye A (2019). Older persons' experiences of adapting to daily life at home after hospital discharge: a qualitative metasummary. BMC Health Serv Res.

[11] Forster AJ, Clark HD, Menard A, Dupuis N, Chernish R, Chandok N, Khan A, van Walraven C (2004). Adverse events among medical patients after discharge from hospital. CMAJ.

[12] Forster AJ, Murff HJ, Peterson JF, Gandhi TK, Bates DW (2003). The incidence and severity of adverse events affecting patients after discharge from the hospital. Ann Intern Med.

[13] Finn KM, Heffner R, Chang Y, Bazari H, Hunt D, Pickell K, Berube R, Raju S, Farrell E, Iyasere C, Thompson R, O'Malley T, O'Donnell W, Karson A (2011). Improving the discharge process by embedding a discharge facilitator in a resident team. J Hosp Med.

[14] Cutler DM (1995). The incidence of adverse medical outcomes under prospective payment. Econometrica.

[15] Kosecoff J, Kahn KL, Rogers WH, Reinisch EJ, Sherwood MJ, Rubenstein LV, Draper D, Roth CP, Chew C, Brook RH (1990). Prospective payment system and impairment at discharge. The 'quicker-and-sicker' story revisited. JAMA.

[16] Abrashkin KA, Cho HJ, Torgalkar S, Markoff B (2012). Improving transitions of care from hospital to home: what works?. Mt Sinai J Med.

[17] Rennke S, Ranji SR (2015). Transitional care strategies from hospital to home: a review for the neurohospitalist. Neurohospitalist.

[18] Gulliford M, Naithani S, Morgan M (2006). What is 'continuity of care'?. J Health Serv Res Policy.

[19] Goldfield NI, McCullough EC, Hughes JS, Tang AM, Eastman B, Rawlins LK, Averill RF (2008). Identifying potentially preventable readmissions. Health Care Financ Rev.

[20] Mansukhani RP, Bridgeman MB, Candelario D, Eckert LJ (2015). Exploring transitional care: evidence-based strategies for improving provider communication and reducing readmissions. P T.

[21] Norlyk A, Deleuran CL, Martinsen B (2020). Struggles with infrastructures of information concerning hospital-to-home transitions. Br J Community Nurs.

[22] Allen J, Hutchinson AM, Brown R, Livingston PM (2018). User experience and care for older people transitioning from hospital to home: patients' and carers' perspectives. Health Expect.

[23] Coleman EA, Boult C, American Geriatrics Society Health Care Systems Committee (2003). Improving the quality of transitional care for persons with complex care needs. J Am Geriatr Soc.

[24] Naylor MD (2002). Transitional care of older adults. Annu Rev Nurs Res.

[25] Coleman EA, Smith JD, Frank JC, Min SJ, Parry C, Kramer AM (2004). Preparing patients and caregivers to participate in care delivered across settings: the Care Transitions Intervention. J Am Geriatr Soc.

[26] Coleman EA, Parry C, Chalmers S, Min SJ (2006). The care transitions intervention: results of a randomized controlled trial. Arch Intern Med.

[27] Parry C, Min SJ, Chugh A, Chalmers S, Coleman EA (2009). Further application of the care transitions intervention: results of a randomized controlled trial conducted in a fee-for-service setting. Home Health Care Serv Q.

[28] Voss R, Gardner R, Baier R, Butterfield K, Lehrman S, Gravenstein S (2011). The care transitions intervention: translating from efficacy to effectiveness. Arch Intern Med.

[29] Naylor MD, Brooten D, Campbell R, Jacobsen BS, Mezey MD, Pauly MV, Schwartz JS (1999). Comprehensive discharge planning and home follow-up of hospitalized elders: a randomized clinical trial. JAMA.

[30] Wilkinson ST, Aroop P, Richard JC (2011). Impacting readmission rates and patient satisfaction: results of a discharge pharmacist pilot program. Hosp Pharm.

[31] Hansen LO, Greenwald JL, Budnitz T, Howell E, Halasyamani L, Maynard G, Vidyarthi A, Coleman EA, Williams MV (2013). Project BOOST: effectiveness of a multihospital effort to reduce rehospitalization. J Hosp Med.

[32] Kalu ME, Maximos M, Sengiad S, Dal Bello-Haas V (2019). The role of rehabilitation professionals in care transitions for older adults: a scoping review. Phys Occup Ther Geriatr.

[33] Dohan D, Schrag D (2005). Using navigators to improve care of underserved patients: current practices and approaches. Cancer.

[34] Austin LS, Landis CO, Hanger Jr KH (2012). Extending the continuum of care in congestive heart failure: an interactive technology self-management solution. J Nurs Adm.

[35] Bouwsma EV, Bosmans JE, van Dongen JM, Brölmann HA, Anema JR, Huirne JA (2018). Cost-effectiveness of an Internet-based perioperative care programme to enhance postoperative recovery in gynaecological patients: economic evaluation alongside a stepped-wedge cluster-randomised trial. BMJ Open.

[36] Lavu H, McCall NS, Winter JM, Burkhart RA, Pucci M, Leiby BE, Yeo TP, Cannaday S, Yeo CJ (2019). Enhancing patient outcomes while containing costs after complex abdominal operation: a randomized controlled trial of the whipple accelerated recovery pathway. J Am Coll Surg.

[37] Lafaro KJ, Raz DJ, Kim JY, Hite S, Ruel N, Varatkar G, Erhunmwunsee L, Melstrom L, Lee B, Singh G, Fong Y, Sun V (2020). Pilot study of a telehealth perioperative physical activity intervention for older adults with cancer and their caregivers. Support Care Cancer.

[38] Wolf A, Fors A, Ulin K, Thorn J, Swedberg K, Ekman I (2016). An eHealth diary and symptom-tracking tool combined with person-centered care for improving self-efficacy after a diagnosis of acute coronary syndrome: a substudy of a randomized controlled trial. J Med Internet Res.

[39] Choi J, Jacelon CS, Kalmakis KA (2017). Web-based, pictograph-formatted discharge instructions for low-literacy older adults after hip replacement surgery: findings of end-user evaluation of the website. Rehabil Nurs.

[40] Gurwitz JH, Field TS, Ogarek J, Tjia J, Cutrona SL, Harrold LR, Gagne SJ, Preusse P, Donovan JL, Kanaan AO, Reed G, Garber L (2014). An electronic health record-based intervention to increase follow-up office visits and decrease rehospitalization in older adults. J Am Geriatr Soc.

[41] Whitelaw S, Pellegrini DM, Mamas MA, Cowie M, Van Spall HG (2021). Barriers and facilitators of the uptake of digital health technology in cardiovascular care: a systematic scoping review. Eur Heart J Digit Health.

[42] Palacholla RS, Fischer N, Coleman A, Agboola S, Kirley K, Felsted J, Katz C, Lloyd S, Jethwani K (2019). Provider- and patient-related barriers to and facilitators of digital health technology adoption for hypertension management: scoping review. JMIR Cardio.

[43] Singh H, Armas A, Law S, Tang T, Steele Gray C, Cunningham HV, Thombs R, Ellen M, Sritharan J, Nie JX, Plett D, Jarach CM, Thavorn K, Nelson ML (2021). How digital health solutions align with the roles and functions that support hospital to home transitions for older adults: a rapid review study protocol. BMJ Open.

[44] El-Kareh R, Roy C, Williams DH, Poon EG (2012). Impact of automated alerts on follow-up of post-discharge microbiology results: a cluster randomized controlled trial. J Gen Intern Med.

[45] Steele Gray C, Tang T, Armas A, Backo-Shannon M, Harvey S, Kuluski K, Loganathan M, Nie JX, Petrie J, Ramsay T, Reid R, Thavorn K, Upshur R, Wodchis WP, Nelson M (2020). Building a digital bridge to support patient-centered care transitions from hospital to home for older adults with complex care needs: protocol for a co-design, implementation, and evaluation study. JMIR Res Protoc.

[46] Tricco AC, Langlois EV, Straus SE (2017). Rapid reviews to strengthen health policy and systems: a practical guide. World Health Organization.

[47] Bryant SL (2004). The information needs and information seeking behaviour of family doctors. Health Info Libr J.

[48] Linda NS, Phetlhu DR, Klopper HC (2014). Significance of literature when constructing a theory: a selective literature review. Afr J Phys Health Educ Recreat Dance.

[49] (2021). MEDLINE: overview. National Library of Medicine.

[50] Haddaway NR, Collins AM, Coughlin D, Kirk S (2015). The role of Google Scholar in evidence reviews and its applicability to grey literature searching. PLoS One.

[51] Braun V, Clarke V (2006). Using thematic analysis in psychology. Qual Res Psychol.

[52] Haggerty JL, Reid RJ, Freeman GK, Starfield BH, Adair CE, McKendry R (2003). Continuity of care: a multidisciplinary review. BMJ.

[53] Rethlefsen ML, Kirtley S, Waffenschmidt S, Ayala AP, Moher D, Page MJ, Koffel JB, PRISMA-S Group (2021). PRISMA-S: an extension to the PRISMA Statement for Reporting Literature Searches in Systematic Reviews. Syst Rev.

[54] (2020). World Bank Country and Lending Groups. The World Bank.

[55] Iyawa GE, Herselman M, Botha A (2016). Digital health innovation ecosystems: from systematic literature review to conceptual framework. Procedia Comput Sci.

[56] Hoffmann TC, Glasziou PP, Boutron I, Milne R, Perera R, Moher D, Altman DG, Barbour V, Macdonald H, Johnston M, Lamb SE, Dixon-Woods M, McCulloch P, Wyatt JC, Chan AW, Michie S (2014). Better reporting of interventions: template for intervention description and replication (TIDieR) checklist and guide. BMJ.

[57] Acuña Mora M, Moons P, Sparud-Lundin C, Bratt EL, Goossens E (2016). Assessing the level of evidence on transfer and transition in young people with chronic conditions: protocol of a scoping review. Syst Rev.

[58] Manderson B, McMurray J, Piraino E, Stolee P (2012). Navigation roles support chronically ill older adults through healthcare transitions: a systematic review of the literature. Health Soc Care Community.

[59] Farrell TW, Tomoaia-Cotisel A, Scammon DL, Brunisholz K, Kim J, Day J, Gren LH, Wallace S, Gunning K, Tabler J, Magill MK (2015). Impact of an integrated transition management program in primary care on hospital readmissions. J Healthc Qual.

[60] Eaton CK (2018). Social workers, nurses, or both: who is primarily responsible for hospital discharge planning with older adults?. Soc Work Health Care.

[61] Lim WK, Lambert SF, Gray LC (2003). Effectiveness of case management and post-acute services in older people after hospital discharge. Med J Aust.

[62] Balaban RB, Galbraith AA, Burns ME, Vialle-Valentin CE, Larochelle MR, Ross-Degnan D (2015). A patient navigator intervention to reduce hospital readmissions among high-risk safety-net patients: a randomized controlled trial. J Gen Intern Med.

[63] Campagna V, Nelson SA, Krsnak J (2019). Improving care transitions to drive patient outcomes: the triple aim meets the four pillars. Prof Case Manag.

[64] Dedhia P, Kravet S, Bulger J, Hinson T, Sridharan A, Kolodner K, Wright S, Howell E (2009). A quality improvement intervention to facilitate the transition of older adults from three hospitals back to their homes. J Am Geriatr Soc.

[65] Hume AL, Kirwin J, Bieber HL, Couchenour RL, Hall DL, Kennedy AK, LaPointe NM, Burkhardt CD, Schilli K, Seaton T, Trujillo J, Wiggins B, American College of Clinical Pharmacy (2012). Improving care transitions: current practice and future opportunities for pharmacists. Pharmacotherapy.

[66] Rottman-Sagebiel R, Cupples N, Wang CP, Cope S, Pastewait S, Braden H, MacCarthy D, Conde A, Moris M, Gonzalez EY, Espinoza S (2018). A pharmacist-led transitional care program to reduce hospital readmissions in older adults. Fed Pract.

[67] Dukkers van Emden DM, Ros WJ, Berns MP (1999). Transition of care: an evaluation of the role of the discharge liaison nurse in The Netherlands. J Adv Nurs.

[68] Farkas J, Kadivec S, Kosnik M, Lainscak M (2011). Effectiveness of discharge-coordinator intervention in patients with chronic obstructive pulmonary disease: study protocol of a randomized controlled clinical trial. Respir Med.

[69] Fabbre VD, Buffington AS, Altfeld SJ, Shier GE, Golden RL (2011). Social work and transitions of care: observations from an intervention for older adults. J Gerontol Soc Work.

[70] Dilworth-Anderson P, Hilliard TS, Williams S, Palmer MH (2011). A contextual conceptualization on transitions of care for older persons: shaping the direction of care. Annu Rev Gerontol Geriatr.

[71] Gibson AF, Lee C, Crabb S (2015). ‘Take ownership of your condition’: Australian women’s health and risk talk in relation to their experiences of breast cancer. Health Risk Soc.

[72] Yatim F, Cristofalo P, Ferrua M, Girault A, Lacaze M, Di Palma M, Minvielle E (2017). Analysis of nurse navigators' activities for hospital discharge coordination: a mixed method study for the case of cancer patients. Support Care Cancer.

[73] Kowalkowski M, Chou SH, McWilliams A, Lashley C, Murphy S, Rossman W, Papali A, Heffner A, Russo M, Burke L, Gibbs M, Taylor SP, Atrium Health ACORN Investigators (2019). Structured, proactive care coordination versus usual care for Improving Morbidity during Post-Acute Care Transitions for Sepsis (IMPACTS): a pragmatic, randomized controlled trial. Trials.

[74] Meisinger C, Stollenwerk B, Kirchberger I, Seidl H, Wende R, Kuch B, Holle R (2013). Effects of a nurse-based case management compared to usual care among aged patients with myocardial infarction: results from the randomized controlled KORINNA study. BMC Geriatr.

[75] Occelli P, Touzet S, Rabilloud M, Ganne C, Poupon Bourdy S, Galamand B, Debray M, Dartiguepeyrou A, Chuzeville M, Comte B, Turkie B, Tardy M, Luiggi JS, Jacquet-Francillon T, Gilbert T, Bonnefoy M (2016). Impact of a transition nurse program on the prevention of thirty-day hospital readmissions of elderly patients discharged from short-stay units: study protocol of the PROUST stepped-wedge cluster randomised trial. BMC Geriatr.

[76] Foret Giddens J, Tanner E, Frey K, Reider L, Boult C (2009). Expanding the gerontological nursing role in Guided Care. Geriatr Nurs.

[77] Parry C, Coleman EA (2010). Active roles for older adults in navigating care transitions: lessons learned from the care transitions intervention. Open Longev Sci.

[78] Franklin MM, McCoy MA (2017). The transition of care from hospital to home for patients with hypertension. Nurse Pract.

[79] Missel M, Schønau MN, Pedersen JH, Pedersen PU (2015). Transition from hospital to daily life: a pilot study. Rehabil Nurs.

[80] Schnipper JL, Kirwin JL, Cotugno MC, Wahlstrom SA, Brown BA, Tarvin E, Kachalia A, Horng M, Roy CL, McKean SC, Bates DW (2006). Role of pharmacist counseling in preventing adverse drug events after hospitalization. Arch Intern Med.

[81] Young AM, Mudge AM, Banks MD, Rogers L, Demedio K, Isenring E (2018). Improving nutritional discharge planning and follow up in older medical inpatients: Hospital to Home Outreach for Malnourished Elders. Nutr Diet.

[82] Leland NE, Roberts P, De Souza R, Hwa Chang S, Shah K, Robinson M (2019). Care transition processes to achieve a successful community discharge after postacute care: a scoping review. Am J Occup Ther.

[83] Aboumatar H, Naqibuddin M, Chung S, Adebowale H, Bone L, Brown T, Cooper LA, Gurses AP, Knowlton A, Kurtz D, Piet L, Putcha N, Rand C, Roter D, Shattuck E, Sylvester C, Urteaga-Fuentes A, Wise R, Wolff JL, Yang T, Hibbard J, Howell E, Myers M, Shea K, Sullivan J, Syron L, Wang NY, Pronovost P, BREATHE Study Patient Family Partners Group (2017). Better Respiratory Education and Treatment Help Empower (BREATHE) study: methodology and baseline characteristics of a randomized controlled trial testing a transitional care program to improve patient-centered care delivery among chronic obstructive pulmonary disease patients. Contemp Clin Trials.

[84] Scott LB, Gravely S, Sexton TR, Brzostek S, Brown DL (2013). Examining the effect of a patient navigation intervention on outpatient cardiac rehabilitation awareness and enrollment. J Cardiopulm Rehabil Prev.

[85] Gibson MJ, Kelly KA, Kaplan AK (2012). Family caregiving and transitional care: a critical review. Family Caregiver Alliance.

[86] Kwok T, Lum CM, Chan HS, Ma HM, Lee D, Woo J (2004). A randomized, controlled trial of an intensive community nurse-supported discharge program in preventing hospital readmissions of older patients with chronic lung disease. J Am Geriatr Soc.

[87] Falvey JR, Burke RE, Malone D, Ridgeway KJ, McManus BM, Stevens-Lapsley JE (2016). Role of physical therapists in reducing hospital readmissions: optimizing outcomes for older adults during care transitions from hospital to community. Phys Ther.

[88] Acher AW, Campbell-Flohr SA, Brenny-Fitzpatrick M, Leahy-Gross KM, Fernandes-Taylor S, Fisher AV, Agarwal S, Kind AJ, Greenberg CC, Carayon P, Weber SM (2017). Improving patient-centered transitional care after complex abdominal surgery. J Am Coll Surg.

[89] Bryant-Lukosius D, Carter N, Reid K, Donald F, Martin-Misener R, Kilpatrick K, Harbman P, Kaasalainen S, Marshall D, Charbonneau-Smith R, DiCenso A (2015). The clinical effectiveness and cost-effectiveness of clinical nurse specialist-led hospital to home transitional care: a systematic review. J Eval Clin Pract.

[90] Balaban RB, Williams MV (2010). Improving care transitions: hospitalists partnering with primary care. J Hosp Med.

[91] Balaban RB, Zhang F, Vialle-Valentin CE, Galbraith AA, Burns ME, Larochelle MR, Ross-Degnan D (2017). Impact of a patient navigator program on hospital-based and outpatient utilization over 180 days in a safety-net health system. J Gen Intern Med.

[92] Gordils-Perez J, Schneider SM, Gabel M, Trotter KJ (2017). Oncology nurse navigation: development and implementation of a program at a comprehensive cancer center. Clin J Oncol Nurs.

[93] Parker VA, Clark JA, Leyson J, Calhoun E, Carroll JK, Freund KM, Battaglia TA (2010). Patient navigation: development of a protocol for describing what navigators do. Health Serv Res.

[94] Dillworth J, Dickson VV, Mueller A, Shuluk J, Yoon HW, Capezuti E (2016). Nurses' perspectives: hospitalized older patients and end-of-life decision-making. Nurs Crit Care.

[95] Peters P, Fleuren M, Wijkel D (1997). The quality of the discharge planning process: the effect of a liaison nurse. Int J Qual Health Care.

[96] Plant N, Mallitt KA, Kelly PJ, Usherwood T, Gillespie J, Boyages S, Jan S, McNab J, Essue BM, Gradidge K, Maranan N, Ralphs D, Aspin C, Leeder S (2013). Implementation and effectiveness of 'care navigation', coordinated management for people with complex chronic illness: rationale and methods of a randomised controlled trial. BMC Health Serv Res.

[97] Coleman EA, Chugh A, Williams MV, Grigsby J, Glasheen JJ, McKenzie M, Min SJ (2013). Understanding and execution of discharge instructions. Am J Med Qual.

[98] Naylor MD, Bowles KH, Brooten D (2000). Patient problems and advanced practice nurse interventions during transitional care. Public Health Nurs.

[99] Boult C, Wieland GD (2010). Comprehensive primary care for older patients with multiple chronic conditions: "Nobody rushes you through". JAMA.

[100] Damery S, Flanagan S, Combes G (2016). Does integrated care reduce hospital activity for patients with chronic diseases? An umbrella review of systematic reviews. BMJ Open.

[101] Mackavey C (2016). Advanced practice nurse transitional care model promotes healing in wound care. Care Manag J.

[102] McMurray A, Cooper H (2017). The nurse navigator: an evolving model of care. Collegian.

[103] Naylor MD, Bowles KH, McCauley KM, Maccoy MC, Maislin G, Pauly MV, Krakauer R (2013). High-value transitional care: translation of research into practice. J Eval Clin Pract.

[104] Meade CD, Wells KJ, Arevalo M, Calcano ER, Rivera M, Sarmiento Y, Freeman HP, Roetzheim RG (2014). Lay navigator model for impacting cancer health disparities. J Cancer Educ.

[105] Prieto-Centurion V, Basu S, Bracken N, Calhoun E, Dickens C, DiDomenico RJ, Gallardo R, Gordeuk V, Gutierrez-Kapheim M, Hsu LL, Illendula S, Joo M, Kazmi U, Mutso A, Pickard AS, Pittendrigh B, Sullivan JL, Williams M, Krishnan JA (2019). Design of the patient navigator to Reduce Readmissions (PArTNER) study: a pragmatic clinical effectiveness trial. Contemp Clin Trials Commun.

[106] Jeffs L, Kuluski K, Law M, Saragosa M, Espin S, Ferris E, Merkley J, Dusek B, Kastner M, Bell CM (2017). Identifying effective nurse-led care transition interventions for older adults with complex needs using a structured expert panel. Worldviews Evid Based Nurs.

[107] Wright EA, Graham JH, Maeng D, Tusing L, Zaleski L, Martin R, Seipp R, Citsay B, McDonald B, Bolesta K, Chaundy K, Medico CJ, Gunderman S, Leri F, Guza K, Price R, Gregor C, Parry DT (2019). Reductions in 30-day readmission, mortality, and costs with inpatient-to-community pharmacist follow-up. J Am Pharm Assoc (2003).

[108] Warren C, Lemieux AA, Phoenix Bittner N (2019). Excellence in population health: a successful community-based care transitions program model. Prof Case Manag.

[109] Naylor MD, Aiken LH, Kurtzman ET, Olds DM, Hirschman KB (2011). The care span: the importance of transitional care in achieving health reform. Health Aff (Millwood).

[110] Hjelm M, Holst G, Willman A, Bohman D, Kristensson J (2015). The work of case managers as experienced by older persons (75+) with multi-morbidity - a focused ethnography. BMC Geriatr.

[111] Moher D, Liberati A, Tetzlaff J, Altman DG, PRISMA Group (2009). Preferred reporting items for systematic reviews and meta-analyses: the PRISMA statement. PLoS Med.

[112] Amir O, Ben-Gal T, Weinstein JM, Schliamser J, Burkhoff D, Abbo A, Abraham WT (2017). Evaluation of remote dielectric sensing (ReDS) technology-guided therapy for decreasing heart failure re-hospitalizations. Int J Cardiol.

[113] Ammenwerth E, Woess S, Baumgartner C, Fetz B, van der Heidt A, Kastner P, Modre-Osprian R, Welte S, Poelzl G (2015). Evaluation of an integrated telemonitoring surveillance system in patients with coronary heart disease. Methods Inf Med.

[114] Amroze A, Field TS, Fouayzi H, Sundaresan D, Burns L, Garber L, Sadasivam RS, Mazor KM, Gurwitz JH, Cutrona SL (2019). Use of electronic health record access and audit logs to identify physician actions following noninterruptive alert opening: descriptive study. JMIR Med Inform.

[115] Andikyan V, Rezk Y, Einstein MH, Gualtiere G, Leitao Jr MM, Sonoda Y, Abu-Rustum NR, Barakat RR, Basch EM, Chi DS (2012). A prospective study of the feasibility and acceptability of a Web-based, electronic patient-reported outcome system in assessing patient recovery after major gynecologic cancer surgery. Gynecol Oncol.

[116] Arcilla D, Levin D, Sperber M (2019). Transitioning patients to independence. Home Healthc Now.

[117] Avery KN, Richards HS, Portal A, Reed T, Harding R, Carter R, Bamforth L, Absolom K, O'Connell Francischetto E, Velikova G, Blazeby JM (2019). Developing a real-time electronic symptom monitoring system for patients after discharge following cancer-related surgery. BMC Cancer.

[118] Aziz O, Atallah L, Lo B, Gray E, Athanasiou T, Darzi A, Yang GZ (2011). Ear-worn body sensor network device: an objective tool for functional postoperative home recovery monitoring. J Am Med Inform Assoc.

[119] Backman C, Harley A, Kuziemsky C, Mercer J, Peyton L (2020). MyPath to home Web-based application for the geriatric rehabilitation program at Bruyère continuing care: user-centered design and feasibility testing study. JMIR Form Res.

[120] Barken TL, Thygesen E, Söderhamn U (2018). Unlocking the limitations: living with chronic obstructive pulmonary disease and receiving care through telemedicine-a phenomenological study. J Clin Nurs.

[121] Barnason S, Zimmerman L, Schulz P, Pullen C, Schuelke S (2019). Weight management telehealth intervention for overweight and obese rural cardiac rehabilitation participants: a randomised trial. J Clin Nurs.

[122] Bednarski BK, Nickerson TP, You YN, Messick CA, Speer B, Gottumukkala V, Manandhar M, Weldon M, Dean EM, Qiao W, Wang X, Chang GJ (2019). Randomized clinical trial of accelerated enhanced recovery after minimally invasive colorectal cancer surgery (RecoverMI trial). Br J Surg.

[123] Belarmino A, Walsh R, Alshak M, Patel N, Wu R, Hu JC (2019). Feasibility of a mobile health application to monitor recovery and patient-reported outcomes after robot-assisted radical prostatectomy. Eur Urol Oncol.

[124] Bernocchi P, Vanoglio F, Baratti D, Morini R, Rocchi S, Luisa A, Scalvini S (2016). Home-based telesurveillance and rehabilitation after stroke: a real-life study. Top Stroke Rehabil.

[125] Bernocchi P, Scalvini S, Tridico C, Borghi G, Zanaboni P, Masella C, Glisenti F, Marzegalli M (2012). Healthcare continuity from hospital to territory in Lombardy: TELEMACO project. Am J Manag Care.

[126] Boeni F, Hersberger KE, Arnet I (2015). Success of a sustained pharmaceutical care service with electronic adherence monitoring in patient with diabetes over 12 months. BMJ Case Rep.

[127] Book K, Dinkel A, Henrich G, Stuhr C, Peuker M, Härtl K, Brähler E, Herschbach P (2013). The effect of including a 'psychooncological statement' in the discharge summary on patient-physician communication: a randomized controlled trial. Psychooncology.

[128] Bouwsma EV, Huirne JA, van de Ven PM, Vonk Noordegraaf A, Schaafsma FG, Schraffordt Koops SE, van Kesteren PJ, Brölmann HA, Anema JR (2018). Effectiveness of an Internet-based perioperative care programme to enhance postoperative recovery in gynaecological patients: cluster controlled trial with randomised stepped-wedge implementation. BMJ Open.

[129] Campbell KJ, Louie PK, Bohl DD, Edmiston T, Mikhail C, Li J, Khorsand DA, Levine BR, Gerlinger TL (2019). A novel, automated text-messaging system is effective in patients undergoing total joint arthroplasty. J Bone Joint Surg Am.

[130] Carrier G, Cotte E, Beyer-Berjot L, Faucheron JL, Joris J, Slim K, Groupe Francophone de Réhabilitation Améliorée après Chirurgie (GRACE) (2016). Post-discharge follow-up using text messaging within an enhanced recovery program after colorectal surgery. J Visc Surg.

[131] Chang YL, Tsai YF, Hsu CL, Chao YK, Hsu CC, Lin KC (2020). The effectiveness of a nurse-led exercise and health education informatics program on exercise capacity and quality of life among cancer survivors after esophagectomy: a randomized controlled trial. Int J Nurs Stud.

[132] Chen Y, Brennan N, Magrabi F (2010). Is email an effective method for hospital discharge communication? A randomized controlled trial to examine delivery of computer-generated discharge summaries by email, fax, post and patient hand delivery. Int J Med Inform.

[133] Chen C, Li X, Sun L, Cao S, Kang Y, Hong L, Liang Y, You G, Zhang Q (2019). Post-discharge short message service improves short-term clinical outcome and self-care behaviour in chronic heart failure. ESC Heart Fail.

[134] Chiang LC, Chen WC, Dai YT, Ho YL (2012). The effectiveness of telehealth care on caregiver burden, mastery of stress, and family function among family caregivers of heart failure patients: a quasi-experimental study. Int J Nurs Stud.

[135] Cox CE, Hough CL, Carson SS, White DB, Kahn JM, Olsen MK, Jones DM, Somers TJ, Kelleher SA, Porter LS (2018). Effects of a telephone- and Web-based coping skills training program compared with an education program for survivors of critical illness and their family members. A randomized clinical trial. Am J Respir Crit Care Med.

[136] Cox CE, Hough CL, Jones DM, Ungar A, Reagan W, Key MD, Gremore T, Olsen MK, Sanders L, Greeson JM, Porter LS (2019). Effects of mindfulness training programmes delivered by a self-directed mobile app and by telephone compared with an education programme for survivors of critical illness: a pilot randomised clinical trial. Thorax.

[137] Davis C, Bender M, Smith T, Broad J (2015). Feasibility and acute care utilization outcomes of a post-acute transitional telemonitoring program for underserved chronic disease patients. Telemed J E Health.

[138] Day MA, Anthony CA, Bedard NA, Glass NA, Clark CR, Callaghan JJ, Noiseux NO (2018). Increasing perioperative communication with automated mobile phone messaging in total joint arthroplasty. J Arthroplasty.

[139] Dendale P, De Keulenaer G, Troisfontaines P, Weytjens C, Mullens W, Elegeert I, Ector B, Houbrechts M, Willekens K, Hansen D (2012). Effect of a telemonitoring-facilitated collaboration between general practitioner and heart failure clinic on mortality and rehospitalization rates in severe heart failure: the TEMA-HF 1 (TElemonitoring in the MAnagement of Heart Failure) study. Eur J Heart Fail.

[140] DeVito Dabbs A, Song MK, Myers BA, Li R, Hawkins RP, Pilewski JM, Bermudez CA, Aubrecht J, Begey A, Connolly M, Alrawashdeh M, Dew MA (2016). A randomized controlled trial of a mobile health intervention to promote self-management after lung transplantation. Am J Transplant.

[141] DeVon HA, Rankin SH, Paul SM, Ochs AL (2010). Heart Lung.

[142] Dexter C, Bradley B, Williams DH (2013). Online follow-up after total hip replacement: a first case. BMJ Case Rep.

[143] Andrew DG, Puls SE, Guerrero KS (2016). Utilizing information technology to improve transition of care from hospital to home. J Nurs Educ Pract.

[144] Duncan PW, Abbott RM, Rushing S, Johnson AM, Condon CN, Lycan SL, Lutz BJ, Cummings DM, Pastva AM, D'Agostino Jr RB, Stafford JM, Amoroso RM, Jones SB, Psioda MA, Gesell SB, Rosamond WD, Prvu-Bettger J, Sissine ME, Boynton MD, Bushnell CD, COMPASS Investigative Team (2018). COMPASS-CP: an electronic application to capture patient-reported outcomes to develop actionable stroke and transient ischemic attack care plans. Circ Cardiovasc Qual Outcomes.

[145] Dunn AS, Shetreat-Klein A, Berman J, Cho HJ, Stein L, Lewis C, Hamilton S, To S, Francaviglia P, Kannry J (2015). Improving transitions of care for patients on warfarin: the safe transitions anticoagulation report. J Hosp Med.

[146] Evangelista LS, Lee JA, Moore AA, Motie M, Ghasemzadeh H, Sarrafzadeh M, Mangione CM (2015). Examining the effects of remote monitoring systems on activation, self-care, and quality of life in older patients with chronic heart failure. J Cardiovasc Nurs.

[147] Fitzsimmons DA, Thompson J, Bentley CL, Mountain GA (2016). Comparison of patient perceptions of Telehealth-supported and specialist nursing interventions for early stage COPD: a qualitative study. BMC Health Serv Res.

[148] Frail CK, Garza OW, Haas AL (2016). Experience with technology-supported transitions of care to improve medication use. J Am Pharm Assoc (2003).

[149] Gesell SB, Bushnell CD, Jones SB, Coleman SW, Levy SM, Xenakis JG, Lutz BJ, Bettger JP, Freburger J, Halladay JR, Johnson AM, Kucharska-Newton AM, Mettam LH, Pastva AM, Psioda MA, Radman MD, Rosamond WD, Sissine ME, Halls J, Duncan PW (2019). Implementation of a billable transitional care model for stroke patients: the COMPASS study. BMC Health Serv Res.

[150] Gunter RL, Fernandes-Taylor S, Rahman S, Awoyinka L, Bennett KM, Weber SM, Greenberg CC, Kent KC (2018). Feasibility of an image-based mobile health protocol for postoperative wound monitoring. J Am Coll Surg.

[151] Gustavell T, Sundberg K, Segersvärd R, Wengström Y, Langius-Eklöf A (2019). Decreased symptom burden following surgery due to support from an interactive app for symptom management for patients with pancreatic and periampullary cancer. Acta Oncol.

[152] Gustavell T, Langius-Eklöf A, Wengström Y, Segersvärd R, Sundberg K (2019). Development and feasibility of an interactive smartphone app for early assessment and management of symptoms following pancreaticoduodenectomy. Cancer Nurs.

[153] Haynes SC, Tancredi DJ, Tong K, Hoch JS, Ong MK, Ganiats TG, Evangelista LS, Black JT, Auerbach A, Romano PS, Better Effectiveness After Transition–Heart Failure (BEAT-HF) Research Group (2020). Association of adherence to weight telemonitoring with health care use and death: a secondary analysis of a randomized clinical trial. JAMA Netw Open.

[154] Heaton PC, Frede S, Kordahi A, Lowery L, Moorhead B, Kirby J, Kunze N, Luder H (2019). Improving care transitions through medication therapy management: a community partnership to reduce readmissions in multiple health-systems. J Am Pharm Assoc (2003).

[155] Heiney SP, Donevant SB, Arp Adams S, Parker PD, Chen H, Levkoff S (2020). A smartphone app for self-management of heart failure in older African Americans: feasibility and usability study. JMIR Aging.

[156] Hewner S (2014). A population-based care transition model for chronically ill elders. Nurs Econ.

[157] Ho TW, Huang CT, Chiu HC, Ruan SY, Tsai YJ, Yu CJ, Lai F, HINT Study Group (2016). Effectiveness of telemonitoring in patients with chronic obstructive pulmonary disease in Taiwan-a randomized controlled trial. Sci Rep.

[158] Holleck JL, Gunderson CG, Antony SM, Gupta S, Chang JJ, Merchant N, Lin S, Federman DG (2017). The "Hand-in" project: jump-starting communication between inpatient and outpatient providers. South Med J.

[159] Holt JE, Flint EP, Bowers MT (2011). Got the picture? Using mobile phone technology to reinforce discharge instructions. Am J Nurs.

[160] Hu X, Zhu X, Gao L (2014). Intensive nursing care by an electronic followup system to promote secondary prevention after percutaneous coronary intervention: a randomized trial. J Cardiopulm Rehabil Prev.

[161] Jayaram NM, Khariton Y, Krumholz HM, Chaudhry SI, Mattera J, Tang F, Herrin J, Hodshon B, Spertus JA (2017). Impact of telemonitoring on health status. Circ Cardiovasc Qual Outcomes.

[162] Choi J (2016). Effect of pictograph-based discharge instructions on older adults' comprehension and recall: a pilot study. Res Gerontol Nurs.

[163] Jonker LT, Plas M, de Bock GH, Buskens E, van Leeuwen BL, Lahr MM (2021). Remote home monitoring of older surgical cancer patients: perspective on study implementation and feasibility. Ann Surg Oncol.

[164] Kamoen O, Maqueda V, Yperzeele L, Pottel H, Cras P, Vanhooren G, Vanacker P (2020). Stroke coach: a pilot study of a personal digital coaching program for patients after ischemic stroke. Acta Neurol Belg.

[165] Kang YN, Shen HN, Lin CY, Elwyn G, Huang SC, Wu TF, Hou WH (2019). Does a mobile app improve patients' knowledge of stroke risk factors and health-related quality of life in patients with stroke? A randomized controlled trial. BMC Med Inform Decis Mak.

[166] Karapinar-Çarkıt F, van Breukelen BR, Borgsteede SD, Janssen MJ, Egberts AC, van den Bemt PM (2014). Completeness of patient records in community pharmacies post-discharge after in-patient medication reconciliation: a before-after study. Int J Clin Pharm.

[167] Katz MH, Slack R, Bruno M, McMillan J, Fleming JB, Lee JE, Bednarski B, Papadopoulos J, Matin SF (2016). Outpatient virtual clinical encounters after complex surgery for cancer: a prospective pilot study of "TeleDischarge". J Surg Res.

[168] Keeping-Burke L, Purden M, Frasure-Smith N, Cossette S, McCarthy F, Amsel R (2013). Bridging the transition from hospital to home: effects of the VITAL telehealth program on recovery for CABG surgery patients and their caregivers. Res Nurs Health.

[169] Khan D, Fjerbæk A, Andreasen JJ, Thorup CB, Dinesen B (2018). Cardiac surgery patients’ e-health literacy and their use of a digital portal. Health Educ J.

[170] Klement MR, Rondon AJ, McEntee RM, Greenky MR, Austin MS (2019). Web-based, self-directed physical therapy after total knee arthroplasty is safe and effective for most, but not all, patients. J Arthroplasty.

[171] Kogut SJ, Goldstein E, Charbonneau C, Jackson A, Patry G (2014). Improving medication management after a hospitalization with pharmacist home visits and electronic personal health records: an observational study. Drug Healthc Patient Saf.

[172] Lacson R, Desai S, Landman A, Proctor R, Sumption S, Khorasani R (2018). Impact of a health information technology intervention on the follow-up management of pulmonary nodules. J Digit Imaging.

[173] Layton AM, Whitworth J, Peacock J, Bartels MN, Jellen PA, Thomashow BM (2014). Feasibility and acceptability of utilizing a smartphone based application to monitor outpatient discharge instruction compliance in cardiac disease patients around discharge from hospitalization. Int J Telemed Appl.

[174] Ehnbom EC, Raban MZ, Walter SR, Richardson K, Westbrook JI (2014). Do electronic discharge summaries contain more complete medication information? A retrospective analysis of paper versus electronic discharge summaries. Health Inf Manag.

[175] Lin YH, Huang GS, Ho YL, Lou MF (2020). Patient willingness to undergo a two-week free trial of a telemedicine service for coronary artery disease after coronary intervention: a mixed-methods study. J Nurs Manag.

[176] Lindhardt T, Nielsen MH (2017). Older patients' use of technology for a post-discharge nutritional intervention - a mixed-methods feasibility study. Int J Med Inform.

[177] Lowres N, Mulcahy G, Gallagher R, Ben Freedman S, Marshman D, Kirkness A, Orchard J, Neubeck L (2016). Self-monitoring for atrial fibrillation recurrence in the discharge period post-cardiac surgery using an iPhone electrocardiogram. Eur J Cardiothorac Surg.

[178] Luo J, Dong X, Hu J (2019). Effect of nursing intervention via a chatting tool on the rehabilitation of patients after Total hip Arthroplasty. J Orthop Surg Res.

[179] Lyu KX, Zhao J, Wang B, Xiong GX, Yang WQ, Liu QH, Zhu XL, Sun W, Jiang AY, Wen WP, Lei WB (2016). Smartphone application WeChat for clinical follow-up of discharged patients with head and neck tumors: a randomized controlled trial. Chin Med J (Engl).

[180] Madigan E, Schmotzer BJ, Struk CJ, DiCarlo CM, Kikano G, Piña IL, Boxer RS (2013). Home health care with telemonitoring improves health status for older adults with heart failure. Home Health Care Serv Q.

[181] Markle-Reid M, Valaitis R, Bartholomew A, Fisher K, Fleck R, Ploeg J, Salerno J (2020). An integrated hospital-to-home transitional care intervention for older adults with stroke and multimorbidity: a feasibility study. J Comorb.

[182] Martirosov AL, Seitllari K, Kaurala S, MacDonald N (2020). Pharmacist implementation of a transitions of care electronic referral process to provide hand-off between inpatient and outpatient settings. J Am Pharm Assoc (2003).

[183] Mathar H, Fastholm P, Sandholm N (2015). A qualitative study of televideo consultations for COPD patients. Br J Nurs.

[184] McCloskey R, Jarrett P, Stewart C, Keeping-Burke L (2015). Recruitment and retention challenges in a technology-based study with older adults discharged from a geriatric rehabilitation unit. Rehabil Nurs.

[185] McGillion M, Ouellette C, Good A, Bird M, Henry S, Clyne W, Turner A, Ritvo P, Ritvo S, Dvirnik N, Lamy A, Whitlock R, Lawton C, Walsh J, Paterson K, Duquette J, Sanchez Medeiros K, Elias F, Scott T, Mills J, Harrington D, Field M, Harsha P, Yang S, Peter E, Bhavnani S, Devereaux PJ (2020). Postoperative remote automated monitoring and virtual Hospital-to-Home Care system following cardiac and major vascular surgery: user testing study. J Med Internet Res.

[186] Melholt C, Joensson K, Spindler H, Hansen J, Andreasen JJ, Nielsen G, Noergaard A, Tracey A, Thorup C, Kringelholt R, Dinesen BI (2018). Cardiac patients' experiences with a telerehabilitation web portal: implications for eHealth literacy. Patient Educ Couns.

[187] Wang MY, Shen MJ, Wan LH, Mo MM, Wu Z, Li LL, Neidlinger SH (2020). Effects of a comprehensive reminder system based on the health belief model for patients who have had a stroke on health behaviors, blood pressure, disability, and recurrence from baseline to 6 months: a randomized controlled trial. J Cardiovasc Nurs.

[188] Metcalf M, Glazyrine V, Glavin K, Dahlgren A, Michael C, Bechtel M, Bishop D, DeRuyter M, Mirza M, Taylor J, Wyre HW, Hamilton-Reeves JM, Holzbeierlein JM, Lee EK (2019). The feasibility of a health care application in the treatment of patients undergoing radical cystectomy. J Urol.

[189] Moffet H, Tousignant M, Nadeau S, Mérette C, Boissy P, Corriveau H, Marquis F, Cabana F, Ranger P, Belzile EL, Dimentberg R (2015). In-home telerehabilitation compared with face-to-face rehabilitation after total knee arthroplasty: a noninferiority randomized controlled trial. J Bone Joint Surg Am.

[190] Moro Agud M, Menéndez Colino R, Mauleón Ladrero MD, Ruano Encinar M, Díez Sebastián J, Villamañán Bueno E, Herrero Ambrosio A, González Montalvo JI (2016). Analysis of an electronic medication reconciliation and information at discharge programme for frail elderly patients. Int J Clin Pharm.

[191] Mousa AY, Broce M, Monnett S, Davis E, McKee B, Lucas BD (2019). Results of telehealth electronic monitoring for post discharge complications and surgical site infections following arterial revascularization with groin incision. Ann Vasc Surg.

[192] Moy NY, Lee SJ, Chan T, Grovey B, Boscardin WJ, Gonzales R, Pierluissi E (2014). Development and sustainability of an inpatient-to-outpatient discharge handoff tool: a quality improvement project. Jt Comm J Qual Patient Saf.

[193] Nazar H, Brice S, Akhter N, Kasim A, Gunning A, Slight SP, Watson NW (2016). New transfer of care initiative of electronic referral from hospital to community pharmacy in England: a formative service evaluation. BMJ Open.

[194] Newnham HH, Gibbs HH, Ritchie ES, Hitchcock KI, Nagalingam V, Hoiles A, Wallace E, Georgeson E, Holton S (2015). A feasibility study of the provision of a personalized interdisciplinary audiovisual summary to facilitate care transfer care at hospital discharge: Care Transfer Video (CareTV). Int J Qual Health Care.

[195] Nielsen C, Agerskov H, Bistrup C, Clemensen J (2020). Evaluation of a telehealth solution developed to improve follow-up after kidney transplantation. J Clin Nurs.

[196] Nilsson L, Hellström A, Wennerberg C, Ekstedt M, Schildmeijer K (2020). Patients' experiences of using an e-Health tool for self-management support after prostate cancer surgery: a deductive interview study explained through the FITT framework. BMJ Open.

[197] Nundy S, Razi RR, Dick JJ, Smith B, Mayo A, O'Connor A, Meltzer DO (2013). A text messaging intervention to improve heart failure self-management after hospital discharge in a largely African-American population: before-after study. J Med Internet Res.

[198] Ong MK, Romano PS, Edgington S, Aronow HU, Auerbach AD, Black JT, De Marco T, Escarce JJ, Evangelista LS, Hanna B, Ganiats TG, Greenberg BH, Greenfield S, Kaplan SH, Kimchi A, Liu H, Lombardo D, Mangione CM, Sadeghi B, Sadeghi B, Sarrafzadeh M, Tong K, Fonarow GC, Better Effectiveness After Transition–Heart Failure (BEAT-HF) Research Group (2016). Effectiveness of remote patient monitoring after discharge of hospitalized patients with heart failure: the Better Effectiveness After Transition -- Heart Failure (BEAT-HF) randomized clinical trial. JAMA Intern Med.

[199] Ostrovsky A, O'Connor L, Marshall O, Angelo A, Barrett K, Majeski E, Handrus M, Levy J (2016). Predicting 30- to 120-day readmission risk among Medicare fee-for-service patients using nonmedical workers and mobile technology. Perspect Health Inf Manag.

[200] Park KH, Song MR (2017). The effects of postdischarge telephone counseling and short message service on the knee function, activities of daily living, and life satisfaction of patients undergoing total knee replacement. Orthop Nurs.

[201] Pastora-Bernal JM, Martín-Valero R, Barón-López FJ, Moyano NG, Estebanez-Pérez MJ (2018). Telerehabilitation after arthroscopic subacromial decompression is effective and not inferior to standard practice: preliminary results. J Telemed Telecare.

[202] Pavic M, Klaas V, Theile G, Kraft J, Tröster G, Guckenberger M (2020). Feasibility and usability aspects of continuous remote monitoring of health status in palliative cancer patients using wearables. Oncology.

[203] Pavic M, Klaas V, Theile G, Kraft J, Tröster G, Blum D, Guckenberger M (2020). Mobile health technologies for continuous monitoring of cancer patients in palliative care aiming to predict health status deterioration: a feasibility study. J Palliat Med.

[204] Pedone C, Rossi FF, Cecere A, Costanzo L, Antonelli Incalzi R (2015). Efficacy of a physician-led multiparametric telemonitoring system in very old adults with heart failure. J Am Geriatr Soc.

[205] Piau A, Crissey R, Brechemier D, Balardy L, Nourhashemi F (2019). A smartphone Chatbot application to optimize monitoring of older patients with cancer. Int J Med Inform.

[206] Piette JD, Striplin D, Fisher L, Aikens JE, Lee A, Marinec N, Mansabdar M, Chen J, Gregory LA, Kim CS (2020). Effects of accessible health technology and caregiver support posthospitalization on 30-day readmission risk: a randomized trial. Jt Comm J Qual Patient Saf.

[207] Ponce BA, Brabston EW, Zu S, Watson SL, Baker D, Winn D, Guthrie BL, Shenai MB (2016). Telemedicine with mobile devices and augmented reality for early postoperative care. Annu Int Conf IEEE Eng Med Biol Soc.

[208] Prince M, Allen D, Chittenden S, Misuraca J, Hockenberry MJ (2019). Improving transitional care: the role of handoffs and discharge checklists in hematologic malignancies. Clin J Oncol Nurs.

[209] Ramkumar K, Perera MT, Marudanayagam R, Coldham C, Olliff SP, Mayer DA, Bramhall SR, Buckels JA, Mirza DF (2010). A reaudit of specialist-managed liver trauma after establishment of regional referral and management guidelines. J Trauma.

[210] Reed M, Huang J, Brand R, Graetz I, Jaffe MG, Ballard D, Neugebauer R, Fireman B, Hsu J (2020). Inpatient-outpatient shared electronic health records: telemedicine and laboratory follow-up after hospital discharge. Am J Manag Care.

[211] Reider-Demer M, Raja P, Martin N, Schwinger M, Babayan D (2018). Prospective and retrospective study of videoconference telemedicine follow-up after elective neurosurgery: results of a pilot program. Neurosurg Rev.

[212] Requena M, Montiel E, Baladas M, Muchada M, Boned S, López R, Rodríguez-Villatoro N, Juega J, García-Tornel Á, Rodríguez-Luna D, Pagola J, Rubiera M, Molina CA, Ribo M (2019). Farmalarm. Stroke.

[213] Ritchie CS, Houston TK, Richman JS, Sobko HJ, Berner ES, Taylor BB, Salanitro AH, Locher JL (2016). The E-Coach technology-assisted care transition system: a pragmatic randomized trial. Transl Behav Med.

[214] Sabir FR, Tomlinson J, Strickland-Hodge B, Smith H (2019). Evaluating the connect with pharmacy Web-based intervention to reduce hospital readmission for older people. Int J Clin Pharm.

[215] Saleh S, Larsen JP, Bergsåker-Aspøy J, Grundt H (2014). Re-admissions to hospital and patient satisfaction among patients with chronic obstructive pulmonary disease after telemedicine video consultation - a retrospective pilot study. Multidiscip Respir Med.

[216] Santana MJ, Holroyd-Leduc J, Southern DA, Flemons WW, O'Beirne M, Hill MD, Forster AJ, White DE, Ghali WA, e-DCT Team (2017). A randomised controlled trial assessing the efficacy of an electronic discharge communication tool for preventing death or hospital readmission. BMJ Qual Saf.

[217] Scheper H, Derogee R, Mahdad R, van der Wal RJ, Nelissen RG, Visser LG, de Boer MG (2019). A mobile app for postoperative wound care after arthroplasty: ease of use and perceived usefulness. Int J Med Inform.

[218] Schneider MA, Howard KA (2017). Using technology to enhance discharge teaching and improve coping for patients after stroke. J Neurosci Nurs.

[219] Sinha S, Dillon J, Dargar SK, Archambault A, Martin P, Frankel BA, Lee JI, Carmel AS, Safford M (2019). What to expect that you're not expecting: a pilot video education intervention to improve patient self-efficacy surrounding discharge medication barriers. Health Informatics J.

[220] Smith KJ, Handler SM, Kapoor WN, Martich GD, Reddy VK, Clark S (2016). Automated communication tools and computer-based medication reconciliation to decrease hospital discharge medication errors. Am J Med Qual.

[221] Sorknaes AD, Madsen H, Hallas J, Jest P, Hansen-Nord M (2011). Nurse tele-consultations with discharged COPD patients reduce early readmissions--an interventional study. Clin Respir J.

[222] Sorknaes AD, Bech M, Madsen H, Titlestad IL, Hounsgaard L, Hansen-Nord M, Jest P, Olesen F, Lauridsen J, Østergaard B (2013). The effect of real-time teleconsultations between hospital-based nurses and patients with severe COPD discharged after an exacerbation. J Telemed Telecare.

[223] Sui Y, Wang T, Wang X (2020). The impact of WeChat app-based education and rehabilitation program on anxiety, depression, quality of life, loss of follow-up and survival in non-small cell lung cancer patients who underwent surgical resection. Eur J Oncol Nurs.

[224] Sun V, Dumitra S, Ruel N, Lee B, Melstrom L, Melstrom K, Woo Y, Sentovich S, Singh G, Fong Y (2017). Wireless monitoring program of patient-centered outcomes and recovery before and after major abdominal cancer surgery. JAMA Surg.

[225] Sun V, Raz DJ, Ruel N, Chang W, Erhunmwunsee L, Reckamp K, Tiep B, Ferrell B, McCorkle R, Kim JY (2017). A multimedia self-management intervention to prepare cancer patients and family caregivers for lung surgery and postoperative recovery. Clin Lung Cancer.

[226] Tamblyn R, Abrahamowicz M, Buckeridge DL, Bustillo M, Forster AJ, Girard N, Habib B, Hanley J, Huang A, Kurteva S, Lee TC, Meguerditchian AN, Moraga T, Motulsky A, Petrella L, Weir DL, Winslade N (2019). Effect of an electronic medication reconciliation intervention on adverse drug events: a cluster randomized trial. JAMA Netw Open.

[227] Tamblyn R, Winslade N, Lee TC, Motulsky A, Meguerditchian A, Bustillo M, Elsayed S, Buckeridge DL, Couture I, Qian CJ, Moraga T, Huang A (2018). Improving patient safety and efficiency of medication reconciliation through the development and adoption of a computer-assisted tool with automated electronic integration of population-based community drug data: the RightRx project. J Am Med Inform Assoc.

[228] Timmers T, Janssen L, van der Weegen W, Das D, Marijnissen WJ, Hannink G, van der Zwaard BC, Plat A, Thomassen B, Swen JW, Kool RB, Lambers Heerspink FO (2019). The effect of an app for day-to-day postoperative care education on patients with total knee replacement: randomized controlled trial. JMIR Mhealth Uhealth.

[229] Treskes RW, van Winden LA, van Keulen N, van der Velde ET, Beeres SL, Atsma DE, Schalij MJ (2020). Effect of smartphone-enabled health monitoring devices vs regular follow-up on blood pressure control among patients after myocardial infarction: a randomized clinical trial. JAMA Netw Open.

[230] van den Berg M, Crotty M, Liu E, Killington M, Kwakkel G, van Wegen E (2016). Early supported discharge by caregiver-mediated exercises and e-health support after stroke: a proof-of-concept trial. Stroke.

[231] van der Meij E, Anema JR, Leclercq WK, Bongers MY, Consten EC, Schraffordt Koops SE, van de Ven PM, Terwee CB, van Dongen JM, Schaafsma FG, Meijerink WJ, Bonjer HJ, Huirne JA (2018). Personalised perioperative care by e-health after intermediate-grade abdominal surgery: a multicentre, single-blind, randomised, placebo-controlled trial. Lancet.

[232] Vest JR, Kern LM, Silver MD, Kaushal R, HITEC investigators (2015). The potential for community-based health information exchange systems to reduce hospital readmissions. J Am Med Inform Assoc.

[233] Vesterby MS, Pedersen PU, Laursen M, Mikkelsen S, Larsen J, Søballe K, Jørgensen LB (2017). Telemedicine support shortens length of stay after fast-track hip replacement. Acta Orthop.

[234] Vianello A, Fusello M, Gubian L, Rinaldo C, Dario C, Concas A, Saccavini C, Battistella L, Pellizzon G, Zanardi G, Mancin S (2016). Home telemonitoring for patients with acute exacerbation of chronic obstructive pulmonary disease: a randomized controlled trial. BMC Pulm Med.

[235] Villani A, Malfatto G, Compare A, Della Rosa F, Bellardita L, Branzi G, Molinari E, Parati G (2014). Clinical and psychological telemonitoring and telecare of high risk heart failure patients. J Telemed Telecare.

[236] Wade R, Cartwright C, Shaw K (2012). Factors relating to home telehealth acceptance and usage compliance. Risk Manag Healthc Policy.

[237] Wang L, He L, Tao Y, Sun L, Zheng H, Zheng Y, Shen Y, Liu S, Zhao Y, Wang Y (2017). Evaluating a Web-based coaching program using electronic health records for patients with chronic obstructive pulmonary disease in China: randomized controlled trial. J Med Internet Res.

[238] Wang QQ, Zhao J, Huo XR, Wu L, Yang LF, Li JY, Wang J (2018). Effects of a home care mobile app on the outcomes of discharged patients with a stoma: a randomised controlled trial. J Clin Nurs.

[239] Wang J, Tong Y, Jiang Y, Zhu H, Gao H, Wei R, Que X, Gao L (2018). The effectiveness of extended care based on Internet and home care platform for orthopaedics after hip replacement surgery in China. J Clin Nurs.

[240] Wan LH, Zhang XP, You LM, Ruan HF, Chen SX (2018). The efficacy of a comprehensive reminder system to improve health behaviors and blood pressure control in hypertensive ischemic stroke patients: a randomized controlled trial. J Cardiovasc Nurs.

[241] Whitehouse CR, Long JA, Maloney LM, Daniels K, Horowitz DA, Bowles KH (2020). Feasibility of diabetes self-management telehealth education for older adults during transitions in care. Res Gerontol Nurs.

[242] Wilcock M, Hill A, Wynn A, Kelly L (2019). Accuracy of pharmacist electronic discharge medicines review information transmitted to primary care at discharge. Int J Clin Pharm.

[243] Zheng QY, Geng L, Ni M, Sun JY, Ren P, Ji QB, Li JC, Zhang GQ (2019). Modern instant messaging platform for postoperative follow-up of patients after total joint arthroplasty may reduce re-admission rate. J Orthop Surg Res.

[244] Zhou K, Li J, Li X (2019). Effects of cyclic adjustment training delivered via a mobile device on psychological resilience, depression, and anxiety in Chinese post-surgical breast cancer patients. Breast Cancer Res Treat.

[245] Zhou K, Wang W, Zhao W, Li L, Zhang M, Guo P, Zhou C, Li M, An J, Li J, Li X (2020). Benefits of a WeChat-based multimodal nursing program on early rehabilitation in postoperative women with breast cancer: a clinical randomized controlled trial. Int J Nurs Stud.

[246] Khan A, Uddin S, Srinivasan U (2018). Comorbidity network for chronic disease: a novel approach to understand type 2 diabetes progression. Int J Med Inform.

[247] Lyu WB, Gao Y, Cheng KY, Wu R, Zhou WQ (2019). Effect of self-acupoint massage on blood glucose level and quality of life in older adults with type 2 diabetes mellitus: a randomized controlled trial. J Gerontol Nurs.

[248] Gagnon MP, Desmartis M, Labrecque M, Car J, Pagliari C, Pluye P, Frémont P, Gagnon J, Tremblay N, Légaré F (2012). Systematic review of factors influencing the adoption of information and communication technologies by healthcare professionals. J Med Syst.

[249] Charles L, Jensen L, Torti JM, Parmar J, Dobbs B, Tian PG (2020). Improving transitions from acute care to home among complex older adults using the LACE Index and care coordination. BMJ Open Qual.

[250] Aase K, Waring J (2020). Crossing boundaries: establishing a framework for researching quality and safety in care transitions. Appl Ergon.

[251] Nelson ML, Armas A, Thombs R, Singh H, Fulton J, Cunningham HV, Munce S, Hitzig S, Bettger JP (2021). Synthesising evidence regarding hospital to home transitions supported by volunteers of third sector organisations: a scoping review protocol. BMJ Open.

[252] Olsen RM, Hellzén O, Skotnes LH, Enmarker I (2014). Breakdown in informational continuity of care during hospitalization of older home-living patients: a case study. Int J Integr Care.

[253] Coleman GW, Gibson L, Hanson VL, Bobrowicz A, McKay A (2010). Engaging the disengaged: how do we design technology for digitally excluded older adults?. Proceedings of the 8th ACM Conference on Designing Interactive Systems.

[254] Rogers WA, Fisk AD (2010). Toward a psychological science of advanced technology design for older adults. J Gerontol B Psychol Sci Soc Sci.

[255] Mannheim I, Schwartz E, Xi W, Buttigieg SC, McDonnell-Naughton M, Wouters EJ, van Zaalen Y (2019). Inclusion of older adults in the research and design of digital technology. Int J Environ Res Public Health.

[256] Tappen RM, Cooley ME, Luckmann R, Panday S (2022). Digital health information disparities in older adults: a mixed methods study. J Racial Ethn Health Disparities.

[257] Jiang X, Ming WK, You JH (2019). The cost-effectiveness of digital health interventions on the management of cardiovascular diseases: systematic review. J Med Internet Res.

[258] Waffenschmidt S, Knelangen M, Sieben W, Bühn S, Pieper D (2019). Single screening versus conventional double screening for study selection in systematic reviews: a methodological systematic review. BMC Med Res Methodol.

[259] Hong QN, Pluye P, Fàbregues S, Bartlett G, Boardman F, Cargo M, Dagenais P, Gagnon MP, Griffiths F, Nicolau B, O'Cathain A, Rousseau MC, Vedel I (2019). Improving the content validity of the mixed methods appraisal tool: a modified e-Delphi study. J Clin Epidemiol.

[260] Hong QN, Pluye P, Fàbregues S, Bartlett G, Boardman F, Cargo M, Dagenais P, Gagnon MP, Griffiths F, Nicolau B, O'Cathain A, Rousseau MC, Vedel I (2018). Mixed methods appraisal tool (MMAT), version 2018: user guide. Department of Family Medicine, McGill University.

