# Supplementary material for: Recommendations for the Design and Delivery of Transitions-Focused Digital Health Interventions: Rapid Review
Source: JMIR Aging. 2022 May 19;5(2):e35929. doi: 10.2196/35929 (PMC9164100; doi:10.2196/35929)
Supplement: Multimedia Appendix 1 [file aging_v5i2e35929_app1.docx]

Appendix A: Full search strategy

**Database: Ovid MEDLINE: Epub Ahead of Print, In-Process & Other Non-Indexed Citations, Ovid MEDLINE® Daily and Ovid MEDLINE®**

1 exp "Continuity of Patient Care"/

2 ((care or caring) adj3 (continu* or seamless)).ti,ab,kf.

3 (post-discharge* or post discharge*).ti,ab,kf.

4 (discharge adj3 (patient* or care or coordinat* or protocol* or pathway* or plan* or program* or home* or house* or residence or community or independent or ER or hospital)).ti,ab,kf.

5 (hospital adj4 (home or house or community or residence*)).ti,ab,kf.

6 (transition* adj4 (home or house or community or residence* or care or pathway* or protocol* or healthcare or health care or navigat*)).ti,ab,kf.

7 (home adj3 (return* or transition* or reintegrat* or re-integrat* or follow-up or follow up)).ti,ab,kf.

8 exp Self Care/

9 (aftercare or after-care).ti,ab,kf.

10 (post-hospital* or posthospital* or after-hospital or after hospital).ti,ab,kf.

11 (community adj4 (integrat* or reintegrat* or re-integrat* or reentry or re-entry or reentrance or re-entrance)).ti,ab,kf.

12 Patient Navigation/

13 (patient adj2 navigat*).ti,ab,kf.

14 or/1-13 [Transition to Home Concept]

15 exp Educational Technology/

16 exp Telecommunications/

17 technology transfer/

18 Culturally Appropriate Technology/

19 technology/

20 Information Technology/

21 exp Medical Records/

22 exp Informatics/

23 exp Communications Media/ 24 exp Management Information Systems/

25 ((communicat* or health* or informat* or comput* or medical or integrat*) adj3 (technol* or system* or applicat* or

process*)).ti,ab,kf.

26 ((informat* or communicat*) adj3 (exchang* or tech*)).ti,ab,kf.

27 (electronic adj3 record*).ti,ab,kf.

28 (ehealth or e-health or electronic health or telehealth or tele-health or telemedicine or tele-medicine or telecommunicat* or tele-communicat* or digital health or videoconferenc* or video-conferenc* or virtual care or teleradio* or tele-radio* or telemetry or mobile app* or smartphone* or informatics or mobile health or mhealth or m-health or software or EHR? Or EMR?).ti,ab,kf.

29 ((virtual or remote or distance or mobile or video) adj3 (consult* or health or healthcare or medicine)).ti,ab,kf.

30 exp computing methodologies/

31 Teleradiology/

32 exp Management Information Systems/

33 or/15-32 [eHealth technology Concept]

34 14 and 33 [Discharge to home AND ehealth technology Concepts]

35 exp Residential Facilities/

36 ((nursing adj (home* or facilit*)) or assisted living or long term care).kf.

37 35 or 36 [Terms pertaining to non-independent living]

38 34 not 35 [Exclude patients in assisted living facilities; population is independent living patients]

39 exp aged/ or exp geriatrics/ or exp geriatric nursing/ or (centarian* or centenarian* or elder* or eldest or frail* or geriatri* or nonagenarian* or octagenarian* or octogenarian* or old age* or older adult* or older age* or older female* or older male* or older man or older men or older patient* or older people or older person* or older population or older subject* or older woman or older women or oldest old* or senior* or senium or septuagenarian* or supercentenarian* or very old*).ti,ab,kf.

40 38 and 39 [Results limited to older adults]

41 40 not ((exp infant/ or exp child/ or adolescent/) not exp adult/) [Remove studies indexed as Child only]

42 exp Meta-Analysis as Topic/

43 Review Literature as Topic/

44 Systematic Review/

45 Systematic Reviews as Topic/

46 (meta analy$ or metaanaly$ or systematic review$ or systematic overview$ or scoping review$ or umbrella review$).ti,ab,kf.

47 (cochrane or embase or psychlit or psyclit or psychinfo of psycinfo or cinahl or cinhal or science citation index or scopus or web of science or bids or cancerlit or ageline).ab.

48 (reference list$ or bibliograph$ or hand-search$ or relevant journal$ or manual search$).ab.

49 (review or editorial or guideline or letter or meta analysis or news* or patient education handout).pt.

50 or/42-49 [Modified SIGN filter to retrieve systematic reviews; modified to expand to other reviews as well as non-journal articles]

51 41 not 50 [Remove secondary studies and non-empirical studies]

52 limit 51 to english language

53 limit 52 to yr="2010 -Current" [Limit to ten most recent years]

54 limit 52 to yr="2000 -Current" [Limit to twenty most recent years]

55 38 and 50 [Discharge to Home Concept + eHealth Tech Concept with secondary reviews excluded -- not limited to older adults]

56 38 not 50 [Discharge to Home Concept + eHealth Tech Concept with secondary reviews excluded -- not limited to older adults]

57 limit 56 to english language

58 limit 57 to yr="2010 -Current" [Limit to ten most recent years -- not limited to older adults]

59 limit 57 to yr="2000 -Current" [Limit to twenty most recent years - not limited to older adults]

60 (afghanistan or albania or algeria or american samoa or angola or "antigua and barbuda" or antigua or barbuda or argentina or armenia or armenian or aruba or azerbaijan or bahrain or bangladesh or barbados or republic of belarus or belarus or byelarus or belorussia or byelorussian or belize or british honduras or benin or dahomey or bhutan or bolivia or "bosnia and herzegovina" or bosnia or herzegovina or botswana or bechuanaland or brazil or brasil or bulgaria or burkina faso or burkina fasso or upper volta or burundi or urundi or cabo verde or cape verde or cambodia or kampuchea or khmer republic or cameroon or cameron or cameroun or central african republic or ubangi shari or chad or chile or china or colombia or comoros or comoro islands or iles comores or mayotte or democratic republic of the congo or democratic republic congo or congo or zaire or costa rica or "cote d’ivoire" or "cote d’ ivoire" or cote divoire or cote d ivoire or ivory coast or croatia or cuba or cyprus or czech republic or czechoslovakia or djibouti or french somaliland or dominica or dominican republic or ecuador or egypt or united arab republic or el salvador or equatorial guinea or spanish guinea or eritrea or estonia or eswatini or swaziland or ethiopia or fiji or gabon or gabonese republic or gambia or "georgia (republic)" or georgian or ghana or gold coast or gibraltar or greece or grenada or guam or guatemala or guinea or guinea bissau or guyana or british guiana or haiti or hispaniola or honduras or hungary or india or indonesia or timor or iran or iraq or isle of man or jamaica or jordan or kazakhstan or kazakh or kenya or "democratic people’s republic of korea" or republic of korea or north korea or south korea or korea or kosovo or kyrgyzstan or kirghizia or kirgizstan or kyrgyz republic or kirghiz or laos or lao pdr or "lao people's democratic republic" or latvia or lebanon or lebanese republic or lesotho or basutoland or liberia or libya or libyan arab jamahiriya or lithuania or macau or macao or "macedonia (republic)" or macedonia or madagascar or malagasy republic or malawi or nyasaland or malaysia or malay federation or malaya federation or maldives or indian ocean islands or indian ocean or mali or malta or micronesia or federated states of micronesia or kiribati or marshall islands or nauru or northern mariana islands or palau or tuvalu or mauritania or mauritius or mexico or moldova or moldovian or mongolia or montenegro or morocco or ifni or mozambique or portuguese east africa or myanmar or burma or namibia or nepal or netherlands antilles or nicaragua or niger or nigeria or oman or muscat or pakistan or panama or papua new guinea or new guinea or paraguay or peru or philippines or philipines or phillipines or phillippines or poland or "polish people's republic" or portugal or portuguese republic or puerto rico or romania or russia or russian federation or ussr or soviet union or union of soviet socialist republics or rwanda or ruanda or samoa or pacific islands or polynesia or samoan islands or navigator island or navigator islands or "sao tome and principe" or saudi arabia or senegal or serbia or seychelles or sierra leone or slovakia or slovak republic or slovenia or melanesia or solomon island or solomon islands or norfolk island or norfolk islands or somalia or south africa or south sudan or sri lanka or ceylon or "saint kitts and nevis" or "st. kitts and nevis" or saint lucia or "st. lucia" or "saint vincent and the grenadines" or saint vincent or "st. vincent" or grenadines or sudan or suriname or surinam or dutch guiana or netherlands guiana or syria or syrian arab republic or tajikistan or tadjikistan or tadzhikistan or tadzhik or tanzania or tanganyika or thailand or siam or timor leste or east timor or togo or togolese republic or tonga or "trinidad and tobago" or trinidad or tobago or tunisia or turkey or "turkey (republic)" or turkmenistan or turkmen or uganda or ukraine or uruguay or uzbekistan or uzbek or vanuatu or new hebrides or venezuela or vietnam or viet nam or middle east or west bank or gaza or palestine or yemen or yugoslavia or zambia or zimbabwe or northern rhodesia or global south or africa south of the sahara or sub-saharan africa or subsaharan africa or africa, central or central africa or africa, northern or north africa or northern africa or magreb or maghrib or sahara or africa, southern or southern africa or africa, eastern or east africa or eastern africa or africa, western or west africa or western africa or west indies or indian ocean islands or caribbean or central america or latin america or "south and central america" or south america or asia, central or central asia or asia, northern or north asia or northern asia or asia, southeastern or southeastern asia or south eastern asia or southeast asia or south east asia or asia, western or western asia or europe, eastern or east europe or eastern europe or developing country or developing countries or developing nation? or developing population? or developing world or less developed countr* or less developed nation? or less developed population? or less developed world or lesser developed countr* or lesser developed nation? or lesser developed population? or lesser developed world or under developed countr* or under developed nation? or under developed population? or under developed world or underdeveloped countr* or underdeveloped nation? or underdeveloped population? or underdeveloped world or middle income countr* or middle income nation? or middle income population? or low income countr* or low income nation? or low income population? or lower income countr* or lower income nation? or lower income population? or underserved countr* or underserved nation? or underserved population? or underserved world or under served countr* or under served nation? or under served population? or under served world or deprived countr* or deprived nation? or deprived population? or deprived world or poor countr* or poor nation? or poor population? or poor world or poorer countr* or poorer nation? or poorer population? or poorer world or developing econom* or less developed econom* or lesser developed econom* or under developed econom* or underdeveloped econom* or middle income econom* or low income econom* or lower income econom* or low gdp or low gnp or low gross domestic or low gross national or lower gdp or lower gnp or lower gross domestic or lower gross national or lmic or lmics or third world or lami countr* or transitional countr* or emerging economies or emerging nation?).ti,ab,sh,kf.

61 54 not 60

62 limit 61 to yr="2010 -Current"

**Database: Embase Classic+Embase**

1 exp hospital discharge/

2 aftercare/

3 self care/

4 self medication/

5 (post-discharge* or post discharge*).ti,ab,kw.

6 ((care or caring) adj3 (continu* or seamless)).ti,ab,kw.

7 (patient* adj3 (handoff or hand-off)).ti,ab,kw.

8 (discharge adj3 (patient* or care or coordinat* or protocol* or pathway* or plan* or program* or home* or house* or residence or community or domicile or abode or dwelling or independent or ER or hospital)).ti,ab,kw.

9 patient care/

10 clinical handover/

11 transitional care/

12 patient care planning/

13 (hospital adj4 (home or house or community or residence* or abode or dwelling or domicile)).ti,ab,kw.

14 (transition* adj4 (home or house or community or residence* or domicile or dwelling or care or pathway* or protocol* or healthcare or health care or navigat*)).ti,ab,kw.

15 (home adj3 (return* or transition* or reintegrat* or re-integrat* or follow-up or follow up)).ti,ab,kw.

16 (aftercare or after-care).ti,ab,kw.

17 (post-hospital* or posthospital* or after-hospital or after hospital).ti,ab,kw.

18 (community adj4 (integrat* or reintegrat* or re-integrat* or reentry or re-entry or reentrance or re-entrance)).ti,ab,kw.

19 or/1-18 [Transition to Home Concept]

20 exp mass communication/

21 exp medical technology/

22 exp computer assisted diagnosis/

23 exp information technology device/

24 exp hospital information system/

25 information technology/

26 electronic medical record system/

27 exp electronic health record/

28 electronic patient record/

29 exp information system/

30 information science/

31 medical informatics/

32 nursing informatics/

33 ((communicat* or health* or informat* or comput* or medical or integrat*) adj3 (technol* or system* or applicat* or process*)).ti,ab,kw.

34 ((informat* or communicat*) adj3 (exchang* or tech*)).ti,ab,kw.

35 (electronic adj3 record*).ti,ab,kw.

36 (ehealth or e-health or electronic health or telehealth or tele-health or telemedicine or tele-medicine or tele-nursing or telenursing or telecommunicat* or tele-communicat* or digital health or videoconferenc* or video-conferenc* or virtual care or teleradio* or teleradio* or telemetry or mobile app* or smartphone* or informatics or mobile health or mhealth or m-health or software or EHR? Or EMR?).ti,ab,kw

37 exp "Continuity of Patient Care"/

38 [(care adj3 (continu* or seamless)).ti,ab,kf.]

39 [(post-discharge* or post discharge*).ti,ab,kf.]

40 [(discharge adj3 (patient* or care or coordinat* or protocol* or pathway* or plan* or program* or home* or house* or residence or community or independent or ER or hospital)).ti,ab,kf.]

41 [(hospital adj4 (home or house or community or residence*)).ti,ab,kf.]

42 [(transition* adj4 (home or house or community or residence* or care or pathway* or protocol* or healthcare or health care or navigat*)).ti,ab,kf.]

43 [(home adj3 (return* or transition* or reintegrat* or re-integrat* or follow-up or follow up)).ti,ab,kf.]

44 exp Self Care/

45 [(aftercare or after-care).ti,ab,kf.]

46 [(post-hospital* or posthospital* or after-hospital or after hospital).ti,ab,kf.]

47 [(community adj4 (integrat* or reintegrat* or re-integrat* or reentry or re-entry or reentrance or re-entrance)).ti,ab,kf.]

48 Patient Navigation/

49 [(patient adj2 navigat*).ti,ab,kf.]

50 or/37-49 [Transition to Home Concept]

51 exp Educational Technology/

52 exp Telecommunications/

53 technology transfer/

54 Culturally Appropriate Technology/

55 technology/

56 Information Technology/

57 exp Medical Records/

58 exp Informatics/

59 exp Communications Media/

60 exp Management Information Systems/

61 [((communicat* or health* or informat* or comput* or medical or integrat*) adj3 (technol* or system* or applicat* or process*)).ti,ab,kf.]

62 [((informat* or communicat*) adj3 (exchang* or tech*)).ti,ab,kf.]

63 [(electronic adj3 record*).ti,ab,kf.]

64 [(ehealth or e-health or electronic health or telehealth or tele-health or telemedicine or tele-medicine or telecommunicat* or telecommunicat* or digital health or videoconferenc* or video-conferenc* or virtual care or teleradio* or tele-radio* or telemetry or mobile app* or smartphone* or informatics or mobile health or mhealth or m-health or software or EHR? or EMR?).ti,ab,kf.]

65 [((virtual or remote or distance or mobile or video) adj3 (consult* or health or healthcare or medicine)).ti,ab,kf.]

66 exp computing methodologies/

67 Teleradiology/

68 exp Management Information Systems/

69 or/51-68 [eHealth technology Concept]

70 50 and 69 [Discharge to home AND ehealth technology Concepts]

71 exp Residential Facilities/

72 [((nursing adj (home* or facilit*)) or assisted living or long term care).kf.]

73 71 or 72 [Terms pertaining to non-independent living]

74 70 not 71 [Exclude patients in assisted living facilities; population is independent living patients]

75 [exp aged/ or exp geriatrics/ or exp geriatric nursing/ or (centarian* or centenarian* or elder* or eldest or frail* or geriatri* or nonagenarian* or octagenarian* or octogenarian* or old age* or older adult* or older age* or older female* or older male* or older man or older men or older patient* or older people or older person* or older population or older subject* or older woman or older women or oldest old* or senior* or senium or septuagenarian* or supercentenarian* or very old*).ti,ab,kf.]

76 74 and 75 [Results limited to older adults]

77 76 not ((exp infant/ or exp child/ or adolescent/) not exp adult/) [Remove studies indexed as Child only]

78 exp Meta-Analysis as Topic/

79 Review Literature as Topic/

80 Systematic Review/

81 Systematic Reviews as Topic/

82 [(meta analy$ or metaanaly$ or systematic review$ or systematic overview$ or scoping review$ or umbrella review$).ti,ab,kf.]

83 (cochrane or embase or psychlit or psyclit or psychinfo of psycinfo or cinahl or cinhal or science citation index or scopus or web of science or bids or cancerlit or ageline).ab.

84 (reference list$ or bibliograph$ or hand-search$ or relevant journal$ or manual search$).ab.

85 (review or editorial or guideline or letter or meta analysis or news* or patient education handout).pt.

86 or/78-85 [Modified SIGN filter to retrieve systematic reviews; modified to expand to other reviews as well as non-journal articles]

87 77 not 86 [Remove secondary studies and non-empirical studies]

88 limit 87 to english language

89 limit 88 to yr="2010 -Current" [Limit to ten most recent years]

90 limit 88 to yr="2000 -Current" [Limit to twenty most recent years]

91 74 and 86 [Discharge to Home Concept + eHealth Tech Concept with secondary reviews excluded -- not limited to older adults]

92 74 not 86 [Discharge to Home Concept + eHealth Tech Concept with secondary reviews excluded -- not limited to older adults]

93 limit 92 to english language

94 limit 93 to yr="2010 -Current" [Limit to ten most recent years -- not limited to older adults]

95 limit 93 to yr="2000 -Current" [Limit to twenty most recent years - not limited to older adults]

96 [(afghanistan or albania or algeria or american samoa or angola or "antigua and barbuda" or antigua or barbuda or argentina or armenia or armenian or aruba or azerbaijan or bahrain or bangladesh or barbados or republic of belarus or belarus or byelarus or belorussia or byelorussian or belize or british honduras or benin or dahomey or bhutan or bolivia or "bosnia and herzegovina" or bosnia or herzegovina or botswana or bechuanaland or brazil or brasil or bulgaria or burkina faso or burkina fasso or upper volta or burundi or urundi or cabo verde or cape verde or cambodia or kampuchea or khmer republic or cameroon or cameron or cameroun or central african republic or ubangi shari or chad or chile or china or colombia or comoros or comoro islands or iles comores or mayotte or democratic republic of the congo or democratic republic congo or congo or zaire or costa rica or "cote d’ivoire" or "cote d’ivoire" or cote divoire or cote d ivoire or ivory coast or croatia or cuba or cyprus or czech republic or czechoslovakia or djibouti or french somaliland or dominica or dominican republic or ecuador or egypt or united arab republic or el salvador or equatorial guinea or spanish guinea or eritrea or estonia or eswatini or swaziland or ethiopia or fiji or gabon or gabonese republic or gambia or "georgia (republic)" or georgian or ghana or gold coast or gibraltar or greece or grenada or guam or guatemala or guinea or guinea bissau or guyana or british guiana or haiti or hispaniola or honduras or hungary or india or indonesia or timor or iran or iraq or isle of man or jamaica or jordan or kazakhstan or kazakh or kenya or "democratic people’s republic of korea" or republic of korea or north korea or south korea or korea or kosovo or kyrgyzstan or kirghizia or kirgizstan or kyrgyz republic or kirghiz or laos or lao pdr or "lao people's democratic republic" or latvia or lebanon or lebanese republic or lesotho or basutoland or liberia or libya or libyan arab jamahiriya or lithuania or macau or macao or "macedonia (republic)" or macedonia or madagascar or malagasy republic or malawi or nyasaland or malaysia or malay federation or malaya federation or maldives or indian ocean islands or indian ocean or mali or malta or micronesia or federated states of micronesia or kiribati or marshall islands or nauru or northern mariana islands or palau or tuvalu or mauritania or mauritius or mexico or moldova or moldovian or mongolia or montenegro or morocco or ifni or mozambique or portuguese east africa or myanmar or burma or namibia or nepal or netherlands antilles or nicaragua or niger or nigeria or oman or muscat or pakistan or panama or papua new guinea or new guinea or paraguay or peru or philippines or philipines or phillipines or phillippines or poland or "polish people's republic" or portugal or portuguese republic or puerto rico or romania or russia or russian federation or ussr or soviet union or union of soviet socialist republics or rwanda or ruanda or samoa or pacific islands or polynesia or samoan islands or navigator island or navigator islands or "sao tome and principe" or saudi arabia or senegal or serbia or seychelles or sierra leone or slovakia or slovak republic or slovenia or melanesia or solomon island or solomon islands or norfolk island or norfolk islands or somalia or south africa or south sudan or sri lanka or ceylon or "saint kitts and nevis" or "st. kitts and nevis" or saint lucia or "st. lucia" or "saint vincent and the grenadines" or saint vincent or "st. vincent" or grenadines or sudan or suriname or surinam or dutch guiana or netherlands guiana or syria or syrian arab republic or tajikistan or tadjikistan or tadzhikistan or tadzhik or tanzania or tanganyika or thailand or siam or timor leste or east timor or togo or togolese republic or tonga or "trinidad and tobago" or trinidad or tobago or tunisia or turkey or "turkey (republic)" or turkmenistan or turkmen or uganda or ukraine or uruguay or uzbekistan or uzbek or vanuatu or new hebrides or venezuela or vietnam or viet nam or middle east or west bank or gaza or palestine or yemen or yugoslavia or zambia or zimbabwe or northern rhodesia or global south or africa south of the sahara or sub-saharan africa or subsaharan africa or africa, central or central africa or africa, northern or north africa or northern africa or magreb or maghrib or sahara or africa, southern or southern africa or africa, eastern or east africa or eastern africa or africa, western or west africa or western africa or west indies or indian ocean islands or caribbean or central america or latin america or "south and central america" or south america or asia, central or central asia or asia, northern or north asia or northern asia or asia, southeastern or southeastern asia or south eastern asia or southeast asia or south east asia or asia, western or western asia or europe, eastern or east europe or eastern europe or developing country or developing countries or developing nation? or developing population? or developing world or less developed countr* or less developed nation? or less developed population? or less developed world or lesser developed countr* or lesser developed nation? or lesser developed population? or lesser developed world or under developed countr* or under developed nation? or under developed population? or under developed world or underdeveloped countr* or underdeveloped nation? or underdeveloped population? or underdeveloped world or middle income countr* or middle income nation? or middle income population? or low income countr* or low income nation? or low income population? or lower income countr* or lower income nation? or lower income population? or underserved countr* or underserved nation? or underserved population? or underserved world or under served countr* or under served nation? or under served population? or under served world or deprived countr* or deprived nation? or deprived population? or deprived world or poor countr* or poor nation? or poor population? or poor world or poorer countr* or poorer nation? or poorer population? or poorer world or developing econom* or less developed econom* or lesser developed econom* or under developed econom* or underdeveloped econom* or middle income econom* or low income econom* or lower income econom* or low gdp or low gnp or low gross domestic or low gross national or lower gdp or lower gnp or lower gross domestic or lower gross national or lmic or lmics or third world or lami countr* or transitional countr* or emerging economies or emerging nation?).ti,ab,sh,kf.] 97 90 not 96

98 exp hospital discharge/

99 aftercare/

100 self care/

101 self medication/

102 (post-discharge* or post discharge*).ti,ab,kw.

103 ((care or caring) adj3 (continu* or seamless)).ti,ab,kw.

104 (patient* adj3 (handoff or hand-off)).ti,ab,kw.

105 (discharge adj3 (patient* or care or coordinat* or protocol* or pathway* or plan* or program* or home* or house* or residence or community or domicile or abode or dwelling or independent or ER or hospital)).ti,ab,kw.

106 patient care/

107 clinical handover/

108 transitional care/

109 patient care planning/

110 (hospital adj4 (home or house or community or residence* or abode or dwelling or domicile)).ti,ab,kw.

111 (transition* adj4 (home or house or community or residence* or domicile or dwelling or care or pathway* or protocol* or healthcare or health care or navigat*)).ti,ab,kw.

112 (home adj3 (return* or transition* or reintegrat* or re-integrat* or follow-up or follow up)).ti,ab,kw.

113 (aftercare or after-care).ti,ab,kw.

114 (post-hospital* or posthospital* or after-hospital or after hospital).ti,ab,kw.

115 (community adj4 (integrat* or reintegrat* or re-integrat* or reentry or re-entry or reentrance or re-entrance)).ti,ab,kw.

116 or/98-115 [Transition to Home Concept]

117 exp mass communication/

118 exp medical technology/ 119 exp computer assisted diagnosis/

120 exp information technology device/

121 exp hospital information system/

122 information technology/

123 electronic medical record system/

124 exp electronic health record/

125 electronic patient record/

126 exp information system/

127 information science/

128 medical informatics/

129 nursing informatics/

130 ((communicat* or health* or informat* or comput* or medical or integrat*) adj3 (technol* or system* or applicat* or process*)).ti,ab,kw.

131 ((informat* or communicat*) adj3 (exchang* or tech*)).ti,ab,kw.

132 (electronic adj3 record*).ti,ab,kw.

133 (ehealth or e-health or electronic health or telehealth or tele-health or telemedicine or tele-medicine or tele-nursing or telenursing or telecommunicat* or tele-communicat* or digital health or videoconferenc* or video-conferenc* or virtual care or teleradio* or teleradio* or telemetry or mobile app* or smartphone* or informatics or mobile health or mhealth or m-health or software or EHR? Or EMR?).ti,ab,kw.

134 ((virtual or remote* or distance or distant or online or mobile or video or asynchronous) adj3 (consult* or health or healthcare or medicine)).ti,ab,kw.

135 digital bridg*.ti,ab,kw.

136 exp telehealth/

137 or/117-136 [eHealth technology Concept]

138 116 and 137 [Discharge to home AND ehealth technology Concepts]

139 long term care/

140 nursing home/

141 assisted living facility/

142 exp institutional care/

143 hospice/

144 exp hospice care/

145 exp terminal care/

146 residential home/

147 residential care/

148 ((nursing adj (home* or facilit*)) or assisted living or long term care).ti,kw.

149 or/139-148 [Terms pertaining to non-independent living] 150 138 not 149 [Exclude patients in assisted living facilities; population is independent living patients]

151 exp aged/ or exp geriatrics/ or exp elderly care/ or (centarian* or centenarian* or elder* or eldest or frail* or geriatri* or nonagenarian* or octagenarian* or octogenarian* or old age* or older adult* or older age* or older female* or older male* or older man or older men or older patient* or older people or older person* or older population or older subject* or older woman or older women or oldest old*or senior* or senium or septuagenarian* or supercentenarian* or very old*).ti,ab,kw.

152 150 and 151 [Results limited to older adults]

153 152 not (exp juvenile/ not exp adult/) [Remove studies indexed as child only]

154 "review"/

155 "systematic review"/

156 "systematic review (topic)"/

157 exp meta analysis/

158 "meta analysis (topic)"/

159 ((systematic or scoping or realist or rapid) adj (review$1 or overview$1)).ti,ab,kw.

160 (cochrane or embase or psychlit or psyclit or psychinfo of psycinfo or cinahl or cinhal or science citation index or scopus or web of science or bids or cancerlit or ageline).ab.

161 (reference list$ or bibliograph$ or hand-search$ or relevant journal$ or manual search$).ab.

162 (meta analy$ or metaanaly$ or systematic review$ or systematic overview$ or scoping review$ or umbrella review$).ti,ab,kw.

163 limit 162 to (conference abstract or conference paper or "conference review")

164 (conference or editorial or letter or review).pt.

165 or/154-164 [Modified SIGN filter to retrieve systematic reviews; modified to expand to other reviews as well as non-journal articles]

166 153 not 165 [Remove secondary studies and non-empirical studies]

167 limit 166 to english language

168 limit 167 to yr="2010 -Current"

CINAHL

S43 S40 NOT S41

S42 S40 NOT S41

S41 TI ( (systematic or scoping or rapid or realist) N2 (review) ) OR PT ( systematic or scoping or rapid or realist )

S40 S34 NOT S39

S39 S35 OR S36 OR S37 OR S38

S38 (MH "Residential Facilities+") Limiters - Published Date: 20000101- 20201231

S37 (MH "Residential Care") Limiters - Published Date: 20000101- 20201231

S36 (MH "Terminal Care+")

S35 (MH "Long Term Care")

S34 S32 NOT S33

S33 PT review

S32 S16 AND S28

S31 S16 AND S28

S30 S16 AND S28

S29 S16 AND S28

S28 S17 OR S18 OR S19 OR S20 OR S21 OR S22 OR S23 OR S24 OR S25 OR S26 OR S27

S27 TI digital bridg* OR AB digital bridg*

S26 TI ( (virtual or remote* or distance or distant or online or mobile or video or asynchronous) N3 (consult* or health or healthcare or medicine) ) OR AB ( (virtual or remote* or distance or distant or online or mobile or video or asynchronous) N3 (consult* or health or healthcare or medicine) )

S25 TI ( ehealth or e-health or electronic health or telehealth or tele-health or telemedicine or tele-medicine or tele-nursing or telenursing or telecommunicat* or telecommunicat* or digital health or videoconferenc* or videoconferenc* or virtual care or teleradio* or tele-radio* or telemetry or mobile app* or smartphone* or informatics or mobile health or mhealth or m-health or software or EHR? or EMR? ) OR AB ( ( ehealth or e-health or electronic health or telehealth or tele-health or telemedicine or tele-medicine or tele-nursing or telenursing or telecommunicat* or telecommunicat* or digital health or videoconferenc* or videoconferenc* or virtual care or teleradio* or tele-radio* or telemetry or mobile app* or smartphone* or informatics or mobile health or mhealth or m-health or software or EHR? or EMR? ))

S24 TI electronic N3 record* OR AB electronic N3 record*

S23 TI ( (informat* or communicat*) N3 (exchang* or tech*) ) OR AB ( (informat* or communicat*) N3 (exchang* or tech*) )

S22 TI ( (communicat* or informat* or comput* or medical or integrat*) N3 (technol* or system* or applicat* or process*) ) OR AB ( (communicat* or informat* or comput* or medical or integrat*) N3 (technol* or system* or applicat* or process*) )

S21 (MH "Technology+")

S20 (MH "Decision Making, Computer Assisted+")

S19 (MH "Informatics+")

S18 (MH "Information Systems+")

S17 (MH "Telecommunications+")

S16 S1 OR S2 OR S3 OR S4 OR S5 OR S6 OR S7 OR S8 OR S9 OR S10 OR S11 OR S12 OR S13 OR S14 OR S15

S15 TI patient N2 navigat* OR AB patient N2 navigat*

S14 (MH "Patient Navigation")

S13 TI ( post-hospital* or posthospital* or after-hospital or after hospital ) OR AB ( post-hospital* or posthospital* or after-hospital or after hospital )

S12 TI ( aftercare or after-care ) OR AB ( aftercare or aftercare )

S11 TI ( home N3 (return* or transition* or reintegrat* or reintegrat* or follow-up or follow up) ) OR AB ( home N3 (return* or transition* or reintegrat* or re-integrat* or follow-up or follow up) )

S10 TI ( transition* N4 (home or house or community or residence* or domicile or dwelling or care or pathway* or protocol* or healthcare or health care or navigat*) ) OR AB ( transition* N4 (home or house or community or residence* or domicile or dwelling or care or pathway* or protocol* or healthcare or health care or navigat*) )

S9 TI ( hospital N4 (home or house or community or residence* or abode or dwelling or domicile) ) OR AB ( hospital N4 (home or house or community or residence* or abode or dwelling or domicile) )

S8 TI ( discharge N3 (patient* or care or coordinat* or protocol* or pathway* or plan* or program* or home* or house* or residence or community or domicile or abode or dwelling or independent or ER or hospital) ) OR AB ( discharge N3 (patient* or care or coordinat* or protocol* or pathway* or plan* or program* or home* or house* or residence or community or domicile or abode or dwelling or independent or ER or hospital) )

S7 TI ( patient* N3 (handoff or hand-off) ) OR AB ( patient* N3 (handoff or hand-off) )

S6 TI ( post-discharge* or post discharge* ) OR AB ( postdischarge* or post discharge* )

S5 TI ( (care or caring) N3 (continu* or seamless) ) OR AB ( (care or caring) N3 (continu* or seamless) )

S4 (MH "Continuity of Patient Care+")

S3 (MH "Self Care+")

S2 (MH "After Care")

S1 (MH "Patient Discharge+")
