# Supplementary material for: Recommendations for the Design and Delivery of Transitions-Focused Digital Health Interventions: Rapid Review
Source: JMIR Aging. 2022 May 19;5(2):e35929. doi: 10.2196/35929 (PMC9164100; doi:10.2196/35929)
Supplement: Multimedia Appendix 2 [file aging_v5i2e35929_app2.docx]

Appendix B: Phase 2 inclusion criteria

| **Inclusion criteria** | **Description/Examples** |
| --- | --- |
| **Available in English** |  |
| **Published > year 2010** |  |
| **Empirical primary study design** | Pilot Studies (e.g. feasibility or Utility studies), Action Research, Case Studies, Ethnography, Evaluation Methods, Evaluation, Research Experiments, Focus Groups, Field Studies, Interviews, Mail Surveys, Mixed Methods Research, Naturalistic Observation, Online Surveys, Participant Observation, Participatory Research, Qualitative Research, Questionnaires Research, Statistical Analysis, Statistical Studies, Telephone Surveys |
| **Population**  Older adults | Mixed samples should include >1 older adult, not necessarily exclusively (i.e. include if >1 older adult included in the sample) |
| **Non-telephonic digital health technologies**: “an improvement in the way healthcare provision is conceived and delivered by healthcare providers through the use of information and communication technologies to monitor and improve the wellbeing and health of patients and to empower patients in the management of their health and that of their families” (1) | Mobile health (mHealth), health information technology (IT) and wearable devices  NOT prosthetics, robotics, medical imaging technology (x-rays, ultrasounds), interventions only using a standard telephone |
| **Transition intervention:** Recruitment is initiated in the hospital, and intervention continues to the home/community | **Hospital:** inpatient hospital-stay (> 1 night in hospital) (e.g. acute care, rehabilitation). Hospital is NOT ambulatory/outpatient/emergency department visits  **Home**. Home is NOT an assisted living facility/nursing home  **Examples**: acute care to home, rehabilitation to home |
| **Setting:** Applied in “high income” countries (2) | Andorra, Antigua and Barbuda, Aruba, Australia, Austria, The Bahamas, Bahrain, Barbados, Belgium, Bermuda, British Virgin Islands, Brunei Darussalam, Canada, Cayman Islands, Channel Islands, Chile, Croatia, Curacao, Cypress, Czech Republic, Denmark, Estonia, Faroe Islands, Finland, France, French Polynesia, Germany, Gibraltar, Greece, Greenland, Guam, Hong Kong, Hungary, Iceland, Ireland, Isle of Man, Israel, Italy, Japan, Korea Rep., Kuwait, Latvia, Liechtenstein, Lithuania, Luxembourg, Macao Sar, China, Malta, Mauritius, Monaco, Nauru, Netherlands, New Caledonia, New Zealand, Northern Mariana Islands, Norway, Oman, Palau, Panama, Poland, Portugal, Puerto Rico, Qatar, Romania, San Marino, Saudi Arabia, Seychelles, Singapore, Sint Maarten, Slovak Republic, Slovenia, Spain, St. Kitts and Nevis, St. Martin, Sweden, Switzerland, Taiwan China, Trinidad and Tobago, Turks & Caicos Islands, United Arab Emirates, United Kingdom, United States, Uruguay, Virgin Islands (U.S) |

1. Iyawa GE, Herselman M, Botha A. Digital Health Innovation Ecosystems: From Systematic Literature Review to Conceptual Framework. Procedia Comput Sci. 2016;100:244-52.

2. The World Bank. World Bank Country and Lending Groups 2020 [Available from: <https://datahelpdesk.worldbank.org/knowledgebase/articles/906519-world-bank-country-and-lending-groups>.
